# Proteomics of multimorbidity progression across cardiometabolic diseases and cancer in a multinational cohort

**DOI:** 10.1186/s12933-026-03263-4

**Published:** 2026-06-20

**Authors:** Michael J. Stein, Vivian Viallon, Michael F. Leitzmann, Marc J. Gunter, Karl Smith-Byrne, Peggy Ler, Fulvio Ricceri, Giovanna Masala, Sara Beigrezaei, Yvonne Koop, Raúl Zamora-Ros, Ana Jiménez-Zabala, Christina M. Lill, Elio Riboli, Pietro Ferrari, Heinz Freisling

**Affiliations:** 1 Department of Epidemiology and Preventive Medicine, University of Regensburg, Regensburg, Germany; 2 Institute of Epidemiology, Helmholtz Zentrum München, German Research Center for Environmental Health (GmbH), Neuherberg, Germany; 3 International Agency for Research on Cancer (IARC), Nutrition and Metabolism Branch, 25 avenue Tony Garnier, CS90627, Lyon, 69366 France; 4 Cancer Epidemiology and Prevention Research Unit, School of Public Health, Imperial College, London, UK; 5 Cancer Epidemiology Unit, Oxford Population Health, University of Oxford, Oxford, UK; 6 Department of Clinical and Biological Sciences, Centre for Biostatistics, Epidemiology, and Public Health (C- BEPH), University of Turin, Turin, Italy; 7 Clinical Epidemiology Unit, Institute for cancer research, prevention and clinical network (ISPRO), Florence, Italy; 8 Department Global Public Health & Bioethics, Julius Center for Health Sciences and Primary Care, University Medical Center Utrecht, Utrecht, the Netherlands; 9 Department Epidemiology and Health Economics, Julius Center for Health Sciences and Primary Care, University Medical Center, Utrecht, the Netherlands; 10 Unit of Nutrition and Cancer, Institut d’Investigació Biomèdica de Bellvitge, Gran Via de l’Hospitalet, 199, 08908 L’Hospitalet de Llobregat, Barcelona España; 11 Ministry of Health of the Basque Government, Sub-Directorate for Public Health and Addictions of Gipuzkoa, San Sebastián, Gipuzkoa Spain; 12 Group of Epidemiology of Chronic and Communicable Diseases, Biogipuzkoa Health Research Institute, San Sebastián, Gipuzkoa Spain

**Keywords:** Multimorbidity, Cancer, Cardiometabolic disease, Proteomics

## Abstract

**Background:**

Multimorbidity, defined here as the co-occurrence of cardiovascular disease (CVD), type 2 diabetes (T2D), and/or cancer is a major public health challenge. However, its underlying biological mechanisms remain unclear, limiting progress toward identifying shared interventional targets.

**Methods:**

We applied large-scale plasma proteomics (SomaScan 7k; 7,289 aptamers) in 13,270 European Prospective Investigation into Cancer and Nutrition (EPIC) participants to identify protein signatures of multimorbidity. We modelled multimorbidity progression as sequential disease transitions, i.e., from the disease-free state at baseline to a first disease and from the first disease to a second disease. Using weighted multivariable Cox regression, we estimated hazard ratios (HR) and 95% confidence intervals (CI) for risk of cancer, CVD, and T2D. Risk associations were replicated using Olink proteomics in UK Biobank (*N* = 44,567).

**Results:**

We identified 422 aptamers associated with more than one disease (FDR-corrected *P* < 0.05), e.g., 265 aptamers were shared between CVD and T2D. Thirty-eight aptamers were associated with multimorbidity progression. Among these, 27 aptamers showed consistent positive associations across sequential disease transitions, including SEMA6A (disease-free to cancer HR: 1.14; 95% CI 1.05, 1.23; cancer to T2D HR: 2.61; 95% CI 1.76, 3.80). Four aptamers showed consistent inverse associations, including NLGN1 (disease-free to T2D HR: 0.72; 95% CI 0.61, 0.84; T2D to cancer HR: 0.57; 95% CI 0.43, 0.75). Nineteen of the identified proteins were also measured in UK Biobank, with broadly consistent associations.

**Conclusions:**

This study identifies candidate proteins that may indicate molecular pathways to multimorbidity of cardiometabolic diseases and cancer. Future studies should evaluate the causal roles of these proteins for targeted interventions and risk stratification.

**Graphical abstract:**

**Supplementary Information:**

The online version contains supplementary material available at 10.1186/s12933-026-03263-4.

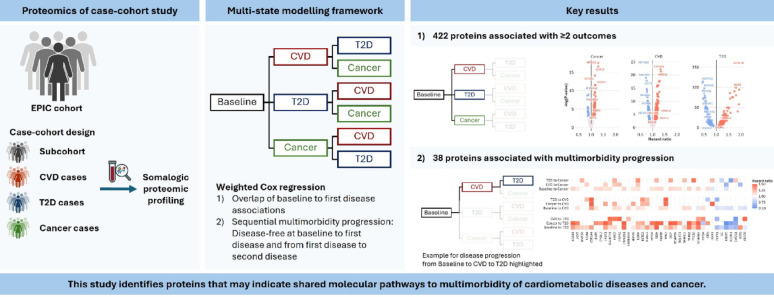

## Research insights

**What is currently known about this topic?**

 Multimorbidity represents a major public health challenge. Cardiovascular diseases, type 2 diabetes, and cancercommonly co-occur. This suggests shared etiology, yet the underlying mechanisms remain unclear.

**What is the key research question?**

 Which circulating proteins are related to multimorbidity of cardiometabolic diseases and cancer?

**What is new?**

 Using a multi-state framework, we identified proteins associated with sequential disease progression. We observed directionally consistent associations across disease transitions. This supports the presence of shared molecular mechanisms underlying multimorbidity.

**How might this study influence clinical practice?**

 Our findings may inform the development of targeted prevention and risk stratification strategies.

## Background

Multimorbidity has emerged as a critical public health challenge [1], driven by the rapidly increasing number of individuals living with multiple chronic conditions over recent decades [2] – a trend projected to continue with population ageing and changing health behaviors [3, 4]. Multimorbidity is linked to functional decline, reduced quality of life, and mortality [5, 6], while the healthcare costs of managing coexisting conditions may exceed the sum of treating individual diseases separately [7]. Despite its growing burden, most medical research and practice guidelines remain centered on individual diseases [3].

Multimorbidity is inherently heterogeneous, characterized by diverse patterns of co-occurring diseases. Broad approaches that aggregate multiple diseases and overlook specific disease combinations may obscure distinct underlying mechanisms [1]. Instead, focusing on commonly co-occurring diseases with shared etiological pathways may offer more precise insights into the mechanisms underlying multimorbidity. Cardiovascular diseases (CVD), type 2 diabetes (T2D), and cancer are among the leading causes of death [8], often co-exist in older adults [9], and share modifiable risk factors such as obesity and physical inactivity [10]. While the individual etiologies of these diseases are well-characterized, their co-occurrence suggests shared biological pathways—several of which align with hallmarks of aging, including chronic inflammation and oxidative stress [11, 12]. A more complete understanding of shared pathways is important for advancing mechanism‑based risk stratification and for developing targeted prevention strategies that can curb multimorbidity in older adults [1].

Progression from an initial chronic disease to multimorbidity is shaped by sociodemographic and lifestyle factors, including sex [13], ethnicity [14], socioeconomic position [15], lifestyle [16, 17], diet [18], and environmental exposures [19], which cumulate which age. Complementing these observations, proteomic studies have begun to reveal molecular contributors. Circulating proteins, influenced by both genetic and non-genetic determinants, provide an integrated readout of systemic biology [20], making them particularly informative for identifying mechanisms shared across chronic diseases. Analyses in UK Biobank identified nine proteins causally linked to CVD among individuals with T2D, largely involved in systemic hormone regulation and IGF receptor binding [21], and 147 proteins as potential predictors of multimorbidity; however, these spanned a heterogeneous spectrum of rare and common diseases rather than focusing on commonly co-occurring diseases, and showed no enrichment in specific mechanistic pathways [22].

Despite growing evidence for shared etiology, comprehensive proteomic profiling across CVD, T2D, and cancer is lacking. To address this research gap, we prospectively applied large-scale proteomics in a multinational cohort to identify circulating proteins and biological pathways shared across these diseases, providing insights into the biology of cardiometabolic and cancer multimorbidity.

## Methods

### Study population and design

The European Prospective Investigation into Cancer and Nutrition (EPIC) is a cohort study of nearly 520,000 participants aged 35 to 74, enrolled across 23 centers across 10 European countries between 1992 and 2000. The study collected data on diet and lifestyle, and participants have provided a blood sample that has been stored in liquid nitrogen [23]. Within the EPIC framework, a multi-endpoint case-cohort study was established, in which blood samples from a total of 17,841 individuals recruited in the UK, the Netherlands, Spain, and Italy underwent proteomic analysis using the SomaScan 7k Assay (SomaLogic, Inc, Boulder, Colorado, USA). This case-cohort study included a subcohort, corresponding to a random subsample of 4,159 participants of the multi-endpoint case-cohort study, a cancer case-component of participants who developed any type of common cancers during follow-up (*n* = 5,713), a CVD case-component of a random sample of participants who developed coronary heart disease and all participants who had a stroke (*n* = 1,200), a T2D case-component of a random sample of participants who developed T2D (*n* = 671), and participants who died (*n* = 3,392) during follow-up.

EPIC was approved by the Ethical Review Boards of the International Agency for Research on Cancer and the Institutional Review Board of each participating EPIC center. Written informed consent was obtained from all participants. This study was approved by the IARC’s Ethics Committee (IEC 25–34).

After exclusion of participants who were selected as neurological disease cases (*n* = 2,737), prevalent diseases (*n* = 729), participants with missing data on incidence diseases (*n* = 526), participants with extreme energy intake/energy requirement status at recruitment (*n* = 302), and missing covariate data (*n* = 277), 13,270 participants were included in the analysis (Supplementary Fig. 1).

### Proteomic measurements

Proteomic profiling was performed using SomaScan reagents (aptamers, *N* = 7,956) following the manufacturer’s protocol [24]. The SomaScan platform employs Slow Off-rate Modified Aptamers (SOMAmers) that specifically bind protein targets and quantify them in relative fluorescence units (RFUs) via DNA microarray. Individual aptamers may bind distinct isoforms of a protein or different sites on the same protein, which can be affected by post-translational modifications or protein complexes, thereby enabling quantification of a broader range of proteins and complexes. RFUs were normalized using hybridization normalization, intraplate median normalization, plate scaling and calibration, and adaptive normalization to a population reference. We excluded aptamers validated for target proteins from non-human organisms, leaving 7,289 human-targeted aptamers for analysis. All protein measurements were performed in baseline blood samples collected prior to any incident event of CVD, T2D, or cancer.

### Ascertainment of cancer, CVD, and T2D cases and multistate framework

In EPIC, incident events of cancer were identified through linkage to population cancer registries and active follow-up. Cancer incidence data were coded according to ICD-O-3 and ICD-10 classifications (ICD-10: C00-C80, excluding non-melanoma skin cancer - C44). Incident CVD diagnoses, including ischemic heart disease (I20-I25), and cerebrovascular diseases (I60-I69), were ascertained through active follow-up via questionnaires, medical records, hospital morbidity registers, contact with medical professionals, and death certificates [25]. T2D diagnoses (E11) were determined from self-reports, primary and secondary care registers, medication use, hospital admissions, and mortality data [26]. Mortality data were obtained at regional or national levels for censoring.

Follow-up commenced at age at recruitment and ended at age at diagnosis of CVD, T2D, or cancer, death, or censoring date (lost or end of follow-up, which was the last center- and event-specific diagnosis date and no later than 31 December 2007), whichever occurred first. Follow-up for incident diseases was structured using a Lexis object, which allows participants’ follow-up time to be split into intervals corresponding to distinct risk periods [27]. Thereby, we modelled each possible transition: from the disease-free state at baseline to a first incident disease and from this first disease to a second incident disease, covering nine transitions in total. This multi-state framework allowed us to investigate multimorbidity progression rather than single disease onset.

### Covariates

Data on socio-demographic and lifestyle factors were collected at recruitment through validated questionnaires, including educational level (none, primary, technical/professional, secondary school, longer education); smoking status (current, past, or never smoker), number of cigarettes currently smoked, average number of cigarettes smoked over their lifetime, the age when participants started and, if applicable, quit smoking (categorized as never, former [quit ≤ 10 years], former [quit 11–20 years], former [quit 20 + years], current [1–15 cigarettes/day], current [16–25 cigarettes/day], current [26 + cigarettes /day], current [pipe/cigar]); adherence to a healthy diet (Mediterranean Diet Score: low, medium, high) [28]; menopausal status in women (premenopausal, perimenopausal, postmenopausal, surgical); and hormone use in postmenopausal women (yes, no). Continuous covariates included alcohol consumption (grams per day); body mass index (BMI), calculated as weight divided by height squared (kg/m²); and weekly leisure-time physical activity (metabolic equivalents of task-hours).

### Cross-platform analyses

We attempted to confirm our findings in an independent cohort. UK Biobank is a prospective cohort that enrolled 502,134 UK participants aged 40 to 69 years between 2006 and 2010. Ethics approval was obtained from the North West Multi-Centre Research Ethics Committee, and all participants provided written informed consent [29]. Extended methods, including proteomic processing and quality assurance, for UK Biobank are provided in the Supplementary Methods.

In brief, UK Biobank generated protein measurements using the Olink Proximity Extension Assay, capturing 2,923 unique proteins in 53,013 participants [30]. About 1,800 protein targets are measured by both Olink and SomaLogic [31]. CVD, T2D, and cancer diagnoses were ascertained from hospital inpatient data, and death from death registries. Covariates included age, sex, country, socioeconomic position, educational level, BMI, physical activity, diet, smoking status, alcohol consumption, sedentary behavior, and previous cancer screening.

### Statistical analyses

**Fig. 1:**
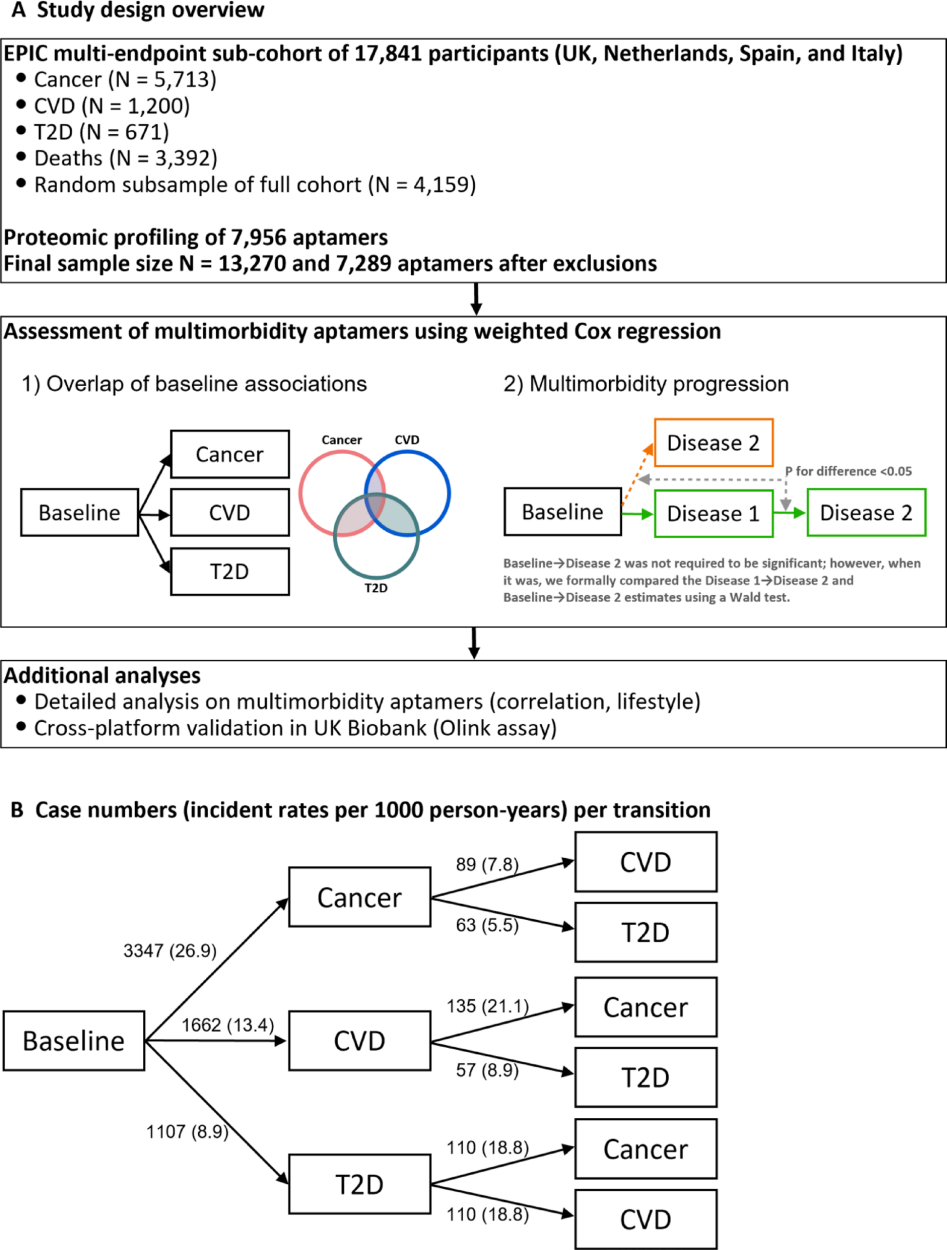
Overview of the study design (**A**) and case numbers with incident rates per 1000 person-years per transition (**B**). CVD, cardiovascular diseases; T2D, type 2 diabetes

We used weighted Cox proportional hazard regression with age as the underlying time metric [32] to estimate hazard ratios (HRs) and corresponding 95% confidence intervals (CIs) for the associations of individual aptamers with CVD, T2D, and cancer (Fig. 1A). Weights were constructed based on each individual’s probability to be included in the study to account for the pre-selection of disease cases [33], and to improve the representativeness of estimates for the underlying source population (Supplementary Methods). We fitted three outcome-specific Cox models for transitions to, in turn, CVD, T2D, and cancer, using robust variance estimation, stratifying by baseline age (5-year groups), sex, and study center, and adjusting for covariates listed above. Each of the three outcome-specific models was based on a stacked Lexis dataset, where each participant contributed one row to three possible transitions, from baseline to the outcome of interest, and from first disease to the outcome of interest. For example, the cancer-specific model included transitions from baseline to cancer, CVD to cancer, and T2D to cancer. Data were augmented and each row contained a start–stop age interval, and a dichotomous event indicator (0/1 = no/event) expressing the onset of the respective specific event. Intervals were censored if another event had occurred, or death, or end of follow-up. Of note, because our objective was etiological inference, we applied cause-specific hazard models rather than subdistribution hazard models, which are more suitable for clinical prediction [34]. For illustration, a participant free of disease at baseline who develops CVD at age t_1_ contributes risk time to the baseline–CVD transition. From age t_1_ onward, if the participant subsequently develops T2D at age t_2_, they further contribute risk time to the CVD–T2D transition.

To obtain transition-specific HRs, each outcome-specific analysis evaluated a main effect of the aptamer on the baseline–outcome transition and an interaction effect between the aptamer and the originating state of that transition. For example, for the cancer-specific analysis, distinct HRs were estimated, i.e., for baseline–cancer, CVD–cancer, and T2D–cancer. P-values for differences were obtained with Wald tests comparing the estimates for CVD–cancer and T2D–cancer with the estimate for baseline–cancer and corrected using the Benjamini–Hochberg false discovery rate (FDR) [35].

First, we assessed the overlap of baseline–outcome transitions across CVD, T2D, and cancer to identify aptamers associated with at least two outcomes (Fig. 1A).

Next, we focused on identifying aptamers with sequential progression to multimorbidity. Aptamers were selected if they fulfilled two requirements. First, the aptamer had to be associated with the transition from the disease-free state at baseline to a first disease. Second, the same aptamer had to be associated with the transition from a first disease to a second disease. This approach was designed to distinguish aptamers that are associated with multiple diseases in an individual from those that simply predict multiple diseases separately. Taking CVD and cancer as an example, we identified aptamers associated with the disease-free–CVD transition as well as the CVD–cancer transition. To ensure that the CVD–cancer association was not merely reflecting a general association with cancer, we additionally required that the log-HR for the CVD–cancer transition exceeded the estimate for the baseline–cancer transition, based on the FDR-corrected Wald tests described above.

In a complementary analysis, we repeated the main analysis but further restricted to aptamers showing an association for progression from the disease-free state to a first disease and subsequently to a second disease, without evidence of a direct disease-free–second disease association. Using the example above, aptamers had to be associated with baseline–CVD and with CVD–cancer, but not with baseline–cancer. This approach allowed us to identify proteins whose association with the second disease was specific to individuals who had developed the first disease, highlighting candidates involved in disease-state-dependent multimorbidity pathways rather than general shared risk.

We conducted several additional analyses to assess the robustness of our findings. First, pairwise correlations between identified aptamers were calculated using Pearson’s correlation coefficients. For strongly correlated aptamers (|r| ≥ 0.5), we ran mutually adjusted models and calculated variance inflation factors (VIF), and assessed HR of these mutually adjusted aptamers. Second, the available subset of identified proteins was assessed in UK Biobank to investigate whether our associations were consistent across cohorts and platforms. Third, we assessed the association between a first incident disease and the risk of a second incident disease by adjusting for time since the first disease onset. Fourth, we conducted lagged analyses (first 1–5 years of follow-up excluded) to assess potential reverse causation. Fifth, in addition to our multivariable-adjusted Cox models, we assessed the contribution of healthy lifestyle factors (BMI, smoking intensity, alcohol intake, diet, and physical activity) to aptamer levels by fitting linear regression models for each identified aptamer, adjusted for age, sex, and study center. Partial R^2^ for each lifestyle factor was computed as the difference in R^2^ between the full model and a reduced model excluding that factor. Finally, to investigate the associated proteins on a pathway level, we performed pathway enrichment analysis using Gene Ontology [36] and the Kyoto Encyclopedia of Genes and Genomes (KEGG) [37]. Enrichment was tested against a background gene set comprising all proteins targeted in EPIC.

All data processing and statistical analyses were performed using R 4.3.1 [38]. Cox regression analyses were performed using the *rms* package [39].

## Results

We analyzed 13,270 participants (56% women; mean age 55 years), among whom 1,662 developed CVD, 1,107 developed T2D, and 3,347 developed cancer as a first incident disease, and 564 subsequently developed a second disease (Fig. 1B). Median follow-up from baseline to first incident disease was 10.1 years (IQR: 6.8–12.4). Median time from first to second disease was 3.1 years (IQR: 1.0–3.9). Participants in the randomly sampled subcohort were more often female, better educated, less likely to smoke, drank less alcohol, and were more physically active than participants from the pre-selected case components (Table 1).

**Table 1:** Baseline characteristics of the case-cohort sub-study in EPIC (*N* = 13,270)

Characteristic	Sub-cohort	Cancer cases	CVD cases	T2D cases	Death cases
N	3,784	5,075	975	645	2,791
*Sex*					
Men	1,423 (37.6)	2,305 (45.4)	504 (51.7)	311 (48.2)	1,296 (46.4)
Women	2,361 (62.4)	2,770 (54.6)	471 (48.3)	334 (51.8)	1,495 (53.6)
Age at recruitment (y)	51.3 (8.6)	55.5 (8.3)	57.3 (8.5)	53.7 (7.7)	60.7 (8.9)
*Education*					
None	673 (17.8)	694 (13.7)	169 (17.3)	146 (22.6)	452 (16.2)
Primary school completed	1,421 (37.6)	1,910 (37.6)	340 (34.9)	273 (42.3)	1,074 (38.5)
Technical/professional school	581 (15.4)	949 (18.7)	212 (21.7)	91 (14.1)	581 (20.8)
Secondary school	555 (14.7)	824 (16.2)	143 (14.7)	83 (12.9)	370 (13.3)
Longer education	554 (14.6)	698 (13.8)	111 (11.4)	52 (8.1)	314 (11.3)
Body mass index (kg/m^2^)	26.9 (4.3)	26.8 (4.2)	27.1 (4.1)	30.5 (4.7)	26.7 (4.4)
Height (cm)	163.8 (8.9)	165.4 (8.9)	165.4 (9.1)	163.7 (9.1)	165.2 (9.1)
*Smoking intensity*					
Never	1,805 (47.7)	1,975 (38.9)	375 (38.5)	274 (42.5)	1,050 (37.6)
Current, 1–15 cigarettes/day	427 (11.3)	590 (11.6)	139 (14.3)	58 (9.0)	316 (11.3)
Current, 16–25 cigarettes/day	278 (7.3)	411 (8.1)	72 (7.4)	57 (8.8)	228 (8.2)
Current, 26 + cigarettes/day	81 (2.1)	167 (3.3)	27 (2.8)	21 (3.3)	93 (3.3)
Current, pipe/cigar	282 (7.5)	464 (9.1)	70 (7.2)	74 (11.5)	173 (6.2)
Former, quit ≤ 10 years	365 (9.6)	576 (11.3)	94 (9.6)	76 (11.8)	285 (10.2)
Former, quit 11–20 years	313 (8.3)	444 (8.7)	90 (9.2)	43 (6.7)	271 (9.7)
Former, quit 20 + years	233 (6.2)	448 (8.8)	108 (11.1)	42 (6.5)	375 (13.4)
Alcohol consumption (g/day)	13.0 (19.8)	14.7 (20.8)	14.4 (22.2)	15.2 (24.5)	13.1 (21.7)
Diet*					
Low	369 (9.8)	766 (15.1)	197 (20.2)	82 (12.7)	632 (22.6)
Medium	1,629 (43.0)	2,219 (43.7)	448 (45.9)	256 (39.7)	1,306 (46.8)
High	1,786 (47.2)	2,090 (41.2)	330 (33.8)	307 (47.6)	853 (30.6)
Physical activity (MET-h/wk)	96.3 (56.5)	92.8 (56.1)	90.9 (55.4)	87.8 (57.6)	90.2 (53.3)

Figures are N (%) or mean (standard deviation)

*Mediterranean diet score in tertiles

CVD, cardiovascular diseases; EPIC, European Prospective Investigation into Cancer and Nutrition; MET, Metabolic equivalents of task; T2D, type 2 diabetes

### Aptamers associated with more than one baseline–outcome transition

**Fig. 2:**
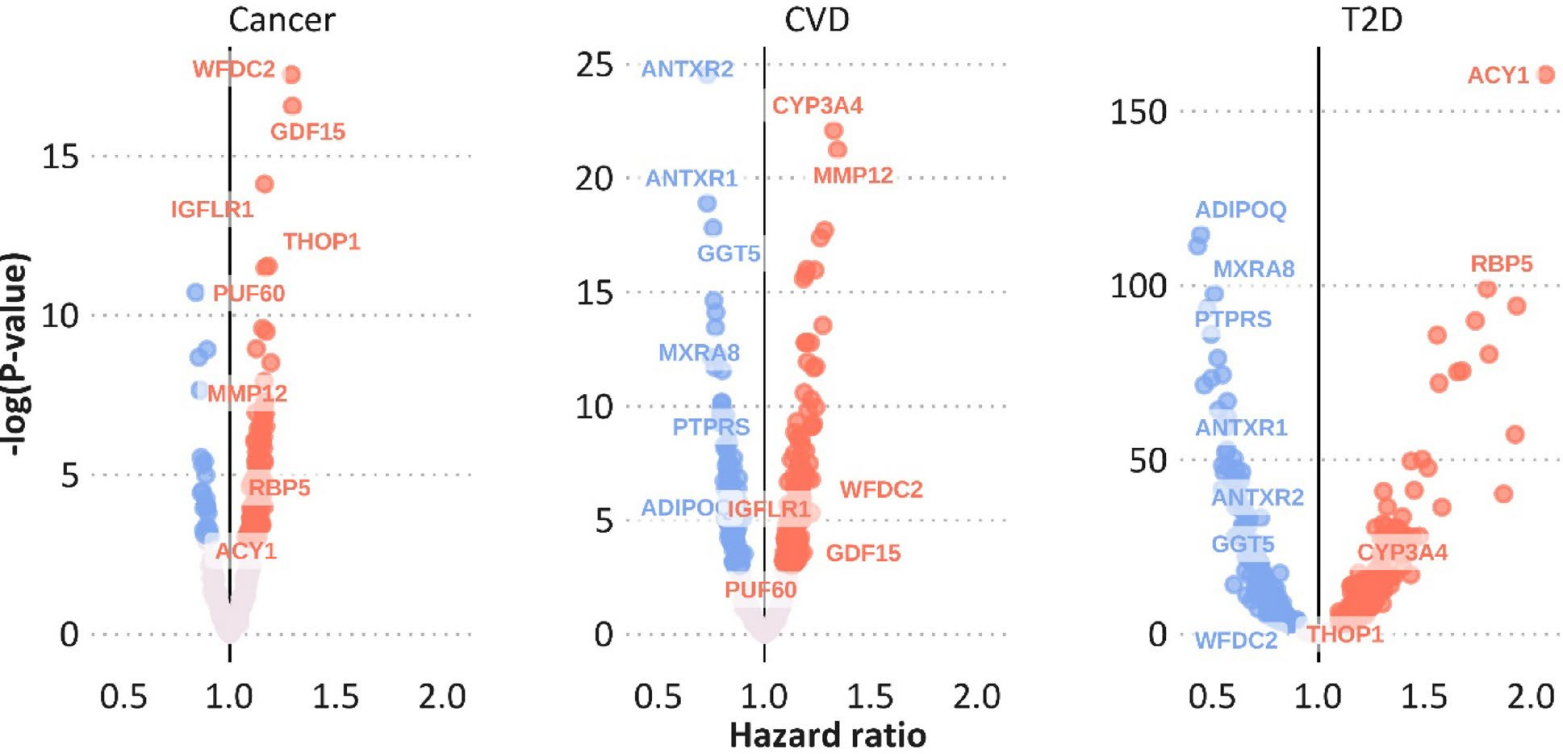
Vulcano plots for aptamers associated with at least two outcomes across cancer, CVD, and T2D. For each outcome (cancer, CVD, T2D), the five most strongly associated aptamers are labelled. These aptamers are also labelled in the other panels to highlight overlap across outcomes. Y-axes display the negative logarithm of false discovery rate-corrected *P*-values. CVD, cardiovascular diseases; T2D, type 2 diabetes

Of 7,289 measured aptamers, 422 were associated with at least two disease-free to first-disease transitions (FDR-corrected *P* < 0.05). Figure 2 displays the associations with at least two outcomes across CVD, T2D, and cancer.

The numbers of statistically significant aptamers shared between two outcomes were 265 for CVD and T2D, 108 for cancer and T2D, 20 for cancer and CVD, and 29 aptamers were associated with all three outcomes. Of the 422 aptamers associated with at least two outcomes, 225 were consistently positively, and 133 were consistently negatively associated.

Of these, the strongest outcome-specific associations included ACY1 for T2D (HR per one standard deviation increase: 2.07; 95% CI 1.91, 2.24; FDR: 1.93 × 10^− 70^), which was also positively associated with overall cancer; ANTXR2 for CVD (HR: 0.73; 95% CI 0.67, 0.79; FDR: 2.20 × 10^− 11^), which was also inversely associated with T2D; and WFDC2 for overall cancer (HR: 1.29; 95% CI 1.19, 1.40; FDR: 2.38 × 10^− 8^), which was also positively associated with CVD, but inversely with T2D (Table 2).

**Table 2:** Hazard ratios and 95% confidence intervals for top baseline associations common across at least two outcomes

Protein	Outcome	Hazard ratio (95% CI)	*P*-value
ACY1	Cancer	1.15 (1.05, 1.26)	3.30 × 10^− 2^
	T2D	2.07 (1.91, 2.24)	1.93 × 10^− 70^
ADIPOQ	CVD	0.83 (0.76, 0.91)	1.28 × 10^− 3^
	T2D	0.45 (0.40, 0.49)	1.72 × 10^− 50^
ANTXR1	CVD	0.73 (0.66, 0.80)	6.20 × 10^− 9^
	T2D	0.56 (0.51, 0.63)	2.46 × 10^− 23^
ANTXR2	CVD	0.73 (0.67, 0.79)	2.20 × 10^− 11^
	T2D	0.64 (0.59, 0.70)	6.05 × 10^− 21^
CYP3A4	CVD	1.33 (1.22, 1.43)	2.55 × 10^− 10^
	T2D	1.32 (1.21, 1.45)	8.67 × 10^− 8^
GDF15	Cancer	1.29 (1.19, 1.41)	6.40 × 10^− 8^
	CVD	1.16 (1.05, 1.28)	3.83 × 10^− 2^
GGT5	CVD	0.76 (0.70, 0.83)	1.85 × 10^− 8^
	T2D	0.72 (0.65, 0.79)	7.62 × 10^− 9^
IGFLR1	Cancer	1.16 (1.10, 1.23)	7.40 × 10^− 7^
	CVD	1.13 (1.05, 1.21)	1.76 × 10^− 2^
MMP12	Cancer	1.16 (1.08, 1.24)	1.16 × 10^− 3^
	CVD	1.34 (1.24, 1.46)	5.97 × 10^− 10^
MXRA8	CVD	0.77 (0.70, 0.84)	1.45 × 10^− 6^
	T2D	0.43 (0.39, 0.48)	4.52 × 10^− 49^
PTPRS	CVD	0.83 (0.76, 0.90)	3.33 × 10^− 4^
	T2D	0.51 (0.46, 0.56)	4.30 × 10^− 43^
PUF60	Cancer	1.17 (1.10, 1.24)	1.03 × 10^− 5^
	CVD	1.12 (1.03, 1.21)	4.65 × 10^− 2^
RBP5	Cancer	1.16 (1.07, 1.26)	4.53 × 10^− 3^
	T2D	1.79 (1.66, 1.94)	9.40 × 10^− 44^
THOP1	Cancer	1.18 (1.11, 1.26)	9.72 × 10^− 6^
	T2D	1.18 (1.09, 1.28)	7.50 × 10^− 4^
WFDC2	Cancer	1.29 (1.19, 1.40)	2.38 × 10^− 8^
	CVD	1.22 (1.11, 1.34)	1.12 × 10^− 3^
	T2D	0.81 (0.72, 0.91)	7.45 × 10^− 3^

*P*-values were false discovery rate-corrected. The table contains the top five aptamers for each outcome

CI, confidence interval; CVD, cardiovascular disease; T2D, type 2 diabetes.

### Aptamers associated with multimorbidity progression

**Fig. 3:**
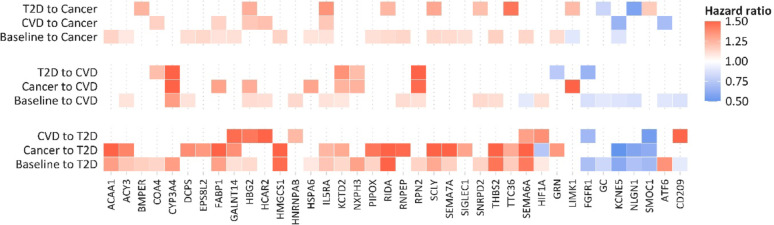
Hazard ratios for the 38 aptamers associated with multimorbidity progression. CVD, cardiovascular diseases; T2D, type 2 diabetes

Thirty-eight aptamers were associated with multimorbidity progression, i.e., they were associated with a first outcome and following this first disease with a second outcome. Of these, 27 showed consistent positive associations, four consistent inverse associations, and seven mixed directions. Figure 3 shows these cross-transition patterns (numeric results provided in the Supplementary Data file).

The strongest consistent positive example was SEMA6A, with a modest association with incident cancer at baseline (HR: 1.14; 95% CI 1.05, 1.23) but a larger association for the transition from cancer to T2D (HR: 2.61; 95% CI 1.76, 3.86; P for difference for baseline–T2D: 9.0 × 10^− 3^). RIDA and HMGCS1 followed a similar pattern. We observed analogous transition-specific differences in non-cancer pathways. For example, RPN2 and CTP3A4 were associated with T2D from the disease-free state and showed larger associations for the transition from T2D to CVD.

Among the strongest inverse associations was NLGN1, which was inversely associated with baseline–T2D (HR: 0.72; 95% CI 0.61, 0.84) and with T2D–cancer (HR: 0.57; 95% CI 0.43, 0.75; P for difference for baseline–cancer: 5.0 × 10^− 4^).

The 38 aptamers showed low to moderate pairwise correlation (median |r| = 0.12, IQR: 0.06–0.15, Supplementary Fig. 2). Four pairs had strong correlations (*r* > 0.5; maximum *r* = 0.74), involving RIDA, SCLY, ACAA1, SEMA6A, SEMA7A, and HMGCS1. In mutual-adjustment models where we included these aptamers within the same model, multicollinearity was low (all VIF < 5): only RIDA, SEMA6A and SEMA7A remained statistically significant for baseline–T2D associations; SCLY remained significantly associated with cancer–T2D (Supplementary Table 1).

### Aptamers associated with multimorbidity progression without direct disease-free-second disease association

Sixteen aptamers were uniquely sequence-specific: i.e., they were associated with the second outcome only after a first disease, without a direct disease-free–second outcome association (Fig. 3).

**Fig. 4:**
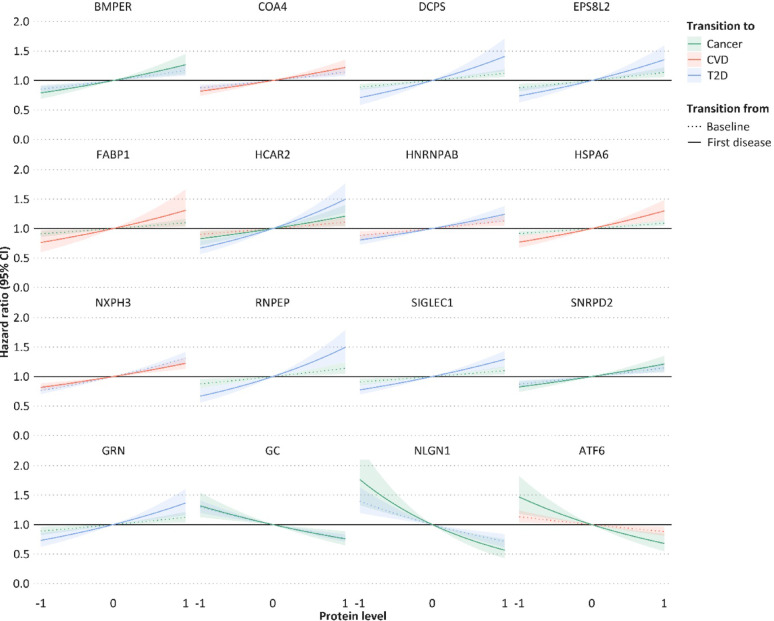
Continuous hazard ratios for the 16 aptamers specifically associated with multimorbidity progression. Dotted lines show the transition from the disease-free state at baseline to a first incident disease; solid lines show the transition from any first incident disease (either cancer, CVD, or T2D) to a second incident disease. CVD, cardiovascular diseases; T2D, type 2 diabetes

When cancer was diagnosed first, DCPS, EPS8L2, GRN, RNPEP, and SIGLEC1 were positively associated with subsequent T2D; and FABP1 and HSPA6 with subsequent CVD. When T2D was first, BMPER, and SNRPD2 were positively associated with subsequent cancer; COA4 and NXPH3 with subsequent CVD. When CVD was first, HCAR2 was positively associated with both subsequent cancer and T2D, and HNRNPAB with subsequent T2D. GC, ATF6, and NLGN1 showed consistent inverse associations across sequences (Fig. 4).

### Associations in UK Biobank

In UK Biobank, protein data were available for 19 of the 38 multimorbidity-related proteins among 44,567 participants (median age 56 years; 54% women). Over a median follow-up of 11.4 years, 11,153 first incident diseases and, during an additional 4.5 years, 2,168 s incident diseases were recorded. Overall, the direction and pattern of associations were broadly comparable to those observed in EPIC, although consistency was mainly seen for disease-free to first disease transitions, particularly for T2D (53–84% consistent direction of effect, 16–68% of associations with FDR < 0.05, Supplementary Fig. 3, Supplementary Data). Second disease transitions were less stable, with 47–74% direction consistency and only < 11% of associations significant in UK Biobank (Supplementary Table 2). Associations for BMPER mirrored the main findings in EPIC. SIGLEC1 was associated with both baseline–T2D and T2D–cancer transitions, albeit in the opposite sequence compared with EPIC (baseline–cancer, cancer–T2D). For some transitions, divergent results were observed for some proteins, including NXPH3 and GC.

### Additional analyses

Additional adjustment for time since the first disease did not influence our results (Supplementary Fig. 4, Supplementary Data). In lagged analyses, risk estimates and 95% CIs remained materially unchanged (Supplementary Table 3). Lastly, the identified aptamers were largely independent of lifestyle. Linear models of lifestyle factors explained little of the proteomic variance for all aptamers, with highest values at 5–6% of variance in FGFR1 and RIDA levels explained by body mass index. All other tested lifestyle factors accounted for negligible proportions of variance (Supplementary Table 4). Gene Ontology enrichment of the 422 proteins associated with more than one outcome showed enrichment for biological processes related to axon development/guidance and cell migration, extracellular and membrane-associated cellular components, and signaling receptor activities, particularly transmembrane receptor tyrosine kinase activity. No significant enrichment was observed in KEGG analyses (Supplementary Table 5).

## Discussion

In this large-scale proteomic study of 13,270 participants from a multinational cohort, we identified 422 circulating aptamers associated with more than one disease across CVD, T2D, and cancer. The largest overlap was observed between CVD and T2D, suggesting a particularly strong shared proteomic signature across cardiometabolic diseases. Several aptamers showed directionally consistent associations, and 38 were associated with sequential multimorbidity progression. We found a pattern of larger associations after the onset of a first disease. The associations were largely independent of lifestyle factors and analyses in UK Biobank showed broadly comparable directions of association to EPIC but were limited by availability of only 19 of 38 proteins and were largely confined to disease-free to first disease transitions, whereas second disease transitions showed limited consistency. These findings suggest that multimorbidity of cardiometabolic diseases and cancer is influenced by shared molecular perturbations.

Beyond single-disease associations, our analysis showed that several proteins, such as SEMA6A, RIDA, and HMGCS1, were more strongly associated with a second disease in an individual. This pattern suggests that these proteins are not merely markers of initial disease risk but may reflect biological processes that persist or worsen after disease-related changes arise. The findings also suggest that baseline proteomic variation may increase an individual’s likelihood of developing multimorbidity following the onset of a first disease.

Our study supports the hypothesis that the co-occurrence of CVD, T2D, and cancer is driven by common biological processes [11, 12]. Prior evidence points to hyperinsulinemia, which promotes carcinogenesis by increasing bioactive insulin-like growth factor (IGF) and sex steroid availability, through reductions in IGF-binding proteins [40–42] and sex hormone-binding globulin [43]. Furthermore, proinflammatory cytokines contribute to insulin resistance [44] and are implicated in the pathogenesis of both cancer and CVD [45], as well as elevated CVD risk in individuals with T2D [46]. Consistent with, but beyond these established pathways, we identified a large set of 422 circulating proteins common across CVD, T2D, and cancer with largely concordant directions of association. Pathway enrichment highlighted cell–cell communication, adhesion, and migratory signaling, consistent with tissue remodeling processes.

Our findings provide evidence for shared molecular mechanisms underlying cardiometabolic disease progression. The largest overlap was observed between CVD and T2D, and several proteins showed directionally consistent associations across these transitions. Multiple proteins (e.g., anthrax toxin receptors, ADIPOQ, MXRA8) were inversely associated with both CVD and T2D, while others (e.g., RPN2, COA4, HCAR2) were positively associated with progression across CVD and T2D.WFDC2 showed opposite associations with CVD (and cancer) versus T2D, with limited mechanistic characterization in cardiometabolic disease. Higher circulating WFDC2 has recently been associated with multiple incident CVD phenotypes [47], suggesting broad involvement in cardiovascular pathophysiology. In contrast, we observed an inverse association with T2D, with mechanisms that remain incompletely understood.

Among the proteins associated with sequential multimorbidity patterns, several mapped to coherent metabolic and inflammatory pathways. Regarding positively associated proteins, SEMA6A and SEMA7A were positively associated with cancer and subsequent T2D, implicating regulation of cell motility and immune response in multimorbidity [48]. Semaphorins have a known role in cancerogenesis [49] as well as metabolic disorders [50]. FABP1 regulates fatty acid transport and oxidative stress and has been linked to CVD and T2D [51, 52]. BMPER is a marker of visceral adipose tissue [53], and EPS8L2 and RNPEP promote tumor progression [54–56]. In contrast, SIGLEC1 and HCAR2, involved in inflammatory regulation, may represent compensatory responses to vascular and metabolic stress rather than direct causal drivers [57, 58]. Two proteins showed potentially protective associations along the T2D–cancer axis: GC (vitamin D-binding protein), a modulator of immune and inflammatory responses [59], and NLGN1, implicated in endothelial function and diabetic vascular health [60]. ATF6 may exert cardioprotective effects [61] and was inversely associated with CVD and subsequent cancer. The remaining less-characterized proteins, such as HNRNPAB and NXPH3, likely capture additional, unexplored proteomic dimensions of multimorbidity that need further mechanistic investigation.

We additionally examined whether some proteins reflect general susceptibility to multimorbidity or whether their associations are specific to a disease-conditioned context. This was done by identifying proteins associated with progression to a second disease only after onset of a first disease, while showing no association with the same second disease in the disease-free state. This restriction distinguishes proteins likely reflecting general baseline risk from those whose effects emerge only in a modified post-disease state. We identified 16 such proteins with state-dependent associations. For cardiometabolic transitions, examples include COA4, NXPH3, and HNRNPAB. In contrast to proteins associated both with multimorbidity progression and baseline disease risk (e.g., RPN2, CYP3A4), these proteins showed no evidence of association with the second disease in the disease-free state, indicating that their relevance arises only after disease onset. These findings suggest that multimorbidity is not solely driven by shared baseline biology but may also involve disease-dependent biological changes following a first disease that modify susceptibility to subsequent outcomes. This subset of proteins may therefore point to mechanisms particularly relevant for secondary prevention in multimorbidity. The consistency of associations across disease transitions and sensitivity analyses strengthens confidence in the identified proteins as biologically plausible contributors to multimorbidity. Reverse causation remains a possibility, as some proteins may be downstream markers of diseases process rather than causal drivers. However, our associations remained materially unchanged in lagged analyses, excluding the first years of follow-up, supporting their relevance to early disease processes. Further sensitivity analyses suggested that protein levels were largely independent from lifestyle, indicating that our protein–disease associations were not confounded or modified by healthy lifestyle factors. However, confounding by post-baseline lifestyle changes or disease-related behavioral modification remains possible. The relatively low inter-protein correlations indicate that these proteins capture distinct biological processes rather than redundant readouts of the same pathway. Cross-platform analyses in UK Biobank suggested broadly similar directions of protein associations with multimorbidity risk, although consistency was limited and varied across disease sequences. In addition, two previous UK Biobank proteomic studies investigated multimorbidity. One identified a cross-sectional T2D proteomics signature, which was longitudinally associated with coronary heart disease [21], and another reported 147 proteins associated with at least two diseases of any kind [22]. Differences in study design, outcome definitions, and analytical approaches limit direct comparison, but our longitudinal multi-disease framework extends these prior insights. Interpretation of cross-cohort consistency is further complicated by differences between proteomic platforms. SomaScan and Olink assays vary in protein coverage, binding chemistry, and sensitivity to specific isoforms, which may contribute to variability in measured protein levels and effect estimates. Differences in population characteristics and disease ascertainment may further contribute to heterogeneity across cohorts. In this context, concordance in direction of effect may more reliably reflect shared underlying biology than strict statistical replication, and multimorbidity-related proteomic patterns can depend on population context or disease order.

Our study advances understanding of molecular architecture underlying multimorbidity. The identified proteins overlap with known cardiometabolic markers but show disease-state-dependent associations that distinguish them as candidates for multimorbidity signatures. While these associations are biologically plausible, causality cannot be inferred. Future work should integrate causal inference frameworks, such as Mendelian randomization or mediation analyses, and experimental validation to clarify mechanistic pathways. In addition, assessing whether these multimorbidity-related proteins improve clinical risk prediction and early detection of at-risk individuals could provide translational relevance. Future studies in general populations are warranted to evaluate the generalizability of our findings.

### Strengths and limitations

Strengths of our study include a large multi-national cohort with long-term follow-up, providing sufficient power to examine multimorbidity across CVD, T2D, and cancer. High-throughput proteomics enabled comprehensive profiling of circulating proteins. Multiple analytic approaches, including a multi-state framework, sensitivity analyses, and cross-platform validation in an independent cohort, enhanced the robustness and reproducibility of our findings.

Interpretation of our findings should consider several limitations. Aptamer-based measurements may bind multiple isoforms and exhibit cross-reactivity, potentially affecting the specificity of observed associations. While proteins may contribute to multiple diseases via shared pathways, associations with a second disease could also reflect indirect links through a first disease, and correlations between disease-specific proteins may create apparent overlap. We addressed this potential bias using transition-specific and baseline risk-independent analyses as well as protein-protein correlations. Cancer was treated as a single outcome group, which increases heterogeneity across biologically distinct cancer types. This may have attenuated cancer-specific associations and masked subtype-specific protein signatures. Future work is warranted to uncover cancer-type–specific mechanisms. Statistical replication in UK Biobank was limited, although direction of effect was largely consistent across cohorts; some associations may therefore reflect false positives or context-specific effects related to cross-platform and cohort differences. Residual confounding and reliance on single baseline protein measurements also limit causal interpretation. Because protein levels were measured only at baseline, this may have introduced non‑differential measurement error and biased associations toward the null. Further, associations may partly reflect early or subclinical processes related to the first disease rather than purely predisposing mechanisms. Although lagged analyses yielded similar results, reverse causation cannot be fully excluded. While protein measurements preceded disease onset and were not influenced by subsequent treatments, we were unable to account for post-diagnostic treatment pathways that may affect progression from a first to a second disease. Detailed treatment data are not available in EPIC. For second transitions, treatment may act as a mediator or effect modifier; therefore, the estimated risks may capture both biological effects of proteins and treatment-related influences, rather than purely shared etiological mechanisms. Progression to a second disease is conditional on survival after the first event. Although transitions to death were explicitly modeled, estimates for subsequent disease transitions reflect a selected subgroup of survivors and may not be fully generalizable to the entire baseline population. This selection may attenuate associations for second transitions, particularly for diseases with high mortality such as certain cancers. Lastly, the study was restricted to participants from four EPIC countries and was based on a disease-enriched sampling design, which may limit generalizability to the overall EPIC cohort and other populations.

## Conclusion

Our study identified multiple circulating proteins associated with multimorbidity of CVD, T2D, and cancer. For some proteins, associations with the risk of a second disease were stronger than for the first, potentially reflecting molecular alterations associated with increased susceptibility to multimorbidity. These proteins are potential targets for the prevention of multimorbidity. Future research should quantify how proteomic changes evolve across disease stages, establish causal relationships, and evaluate whether these protein signatures can improve early detection or risk prediction of multimorbidity.

## Supplementary Information

Below is the link to the electronic supplementary material.

Supplementary Material 1

Supplementary Material 2

## Data Availability

Data access can be requested via https://epic.iarc.fr/access/index.php. The request will be assessed by the EPIC working groups and the EPIC Steering Committee. After approval by the EPIC Steering Committee, deidentified data will be made available. An agreement will be signed specifying the study protocol, variables, statistical analysis plan, researchers involved, and length of time that the data will be available.UK Biobank is an open access resource. Bona fide researchers can apply to use the UK Biobank dataset by registering and applying at [http://ukbiobank.ac.uk/register-apply/](http:/ukbiobank.ac.uk/register-apply).

## Abbreviations

BMI: Body mass index
CI: Confidence interval
CVD: Cardiovascular diseases
EPIC: European Prospective Investigation into Cancer and Nutrition
FDR: False discovery rate
HR: Hazard ratio
KEGG: Kyoto Encyclopedia of Genes and Genomes
RFU: Relative fluorescence unit
T2D: Type 2 diabetes
VIF: Variance inflation factors
