# Supplementary material for: Proteomics of multimorbidity progression across cardiometabolic diseases and cancer in a multinational cohort
Source: Cardiovasc Diabetol. 2026 Jun 20;25:185. doi: 10.1186/s12933-026-03263-4 (PMC13288577; doi:10.1186/s12933-026-03263-4)
Supplement: Supplementary file 1 — Supplementary Material 1 [file 12933_2026_3263_MOESM1_ESM.docx]

**Proteomics of multimorbidity progression across cardiometabolic diseases and cancer in a multinational cohort**

**Supplementary material**

[Supplementary methods](#_Toc228890125)

[Supplementary Figure 1. Flow chart of participant exclusions](#_Toc228890126)

[Supplementary Figure 2. Pairwise correlation of multimorbidity-associated aptamers](#_Toc228890127)

[Supplementary Figure 3. Cross-platform validation in UK Biobank](#_Toc228890128)

[Supplementary Figure 4. Cox regression with adjustment for time since first disease](#_Toc228890129)

[Supplementary Table 1. Mutual adjustment models of multimorbidity-associated aptamers](#_Toc228890130)

[Supplementary Table 2. Cross-platform confirmation with proportion of consistent effect directions and statistical significance in UK Biobank](#_Toc228890131)

[Supplementary Table 3. Cox regression with first 1-5 years of follow-up excluded](#_Toc228890132)

[Supplementary Table 4. Partial R^2^ analyses for healthy lifestyle factors](#_Toc228890133)

[Supplementary Table 5. Pathways enrichment analysis](#_Toc228890134)

**Corresponding author:** Michael J. Stein, Tel.: +49 89 318748819, Mail: michael.stein@helmholtz-munich.de, Department of Epidemiology and Preventive Medicine, University of Regensburg, Regensburg, Germany

### Supplementary methods

We performed cross-platform validation in UK Biobank. The study collected sociodemographic, lifestyle, and extensive phenotypic data using touchscreen questionnaires, interviews, physical and functional measurements, and biomaterial collection. Ethics approval was obtained from the North West Multi-Centre Research Ethics Committee, and all participants provided written informed consent (1). The UK Biobank Pharma Proteomics Project (UKB-PPP) is a precompetitive consortium that generated protein measurements using the Olink Proximity Extension Assay, capturing 2,923 unique proteins in 53,013 participants. Details of the inclusion process for the UKB-PPP are described elsewhere (2). In brief, an initial sample of 6,229 participants was pre-selected by UKB-PPP members, and the remaining samples were selected from the UK Biobank, ensuring representation of the full cohort in terms of age, sex, and recruitment center. About 1,800 protein targets are measured by both Olink and SomaLogic (3).

The Olink assay technology and the analyses of the UKB-PPP are detailed elsewhere (2, 4). In brief, the relative abundance of 2,923 proteins was quantified using antibodies distributed across four 384-plex panels: inflammation, oncology, cardiometabolic, and neurology. Blood samples were assayed in four 384-well plates consisting of four abundance blocks for each of the four panels per 96 samples, using the Olink Explore platform. This platform is based on proximity extension assays, which are highly sensitive and reproducible, with low cross-reactivity. Relative concentrations of the 2,923 unique proteins were measured by next-generation sequencing and expressed as normalised protein expression (NPX) values on a log-base-2 scale. Following a previously published approach (5), protein values below the limit of detection (LOD) were replaced with the LOD divided by the square root of two (6). No protein had more than 20% missingness. Missing protein expression levels were imputed using k-nearest neighbors (*k*=10), allowing for up to 50% missingness across proteins and 20% per protein. Finally, each protein was rescaled to have a mean of 0 and a standard deviation (SD) of 1.

After exclusion of prevalent cases of cancer, CVD, and T2D (n=7,919) and those with >50% missingness in protein data (n=527), a total of 44,567 participants were included in the analyses.

Participants' vital status was obtained through linkage with health care data and national death registries (7). Follow-up began at baseline and ended at the date of complete follow-up (December 2016 for Wales, December 2020 for England, November 2021 for Scotland) (8), loss to follow-up, or death, whichever came first. Cancer incidence data were coded according to ICD-O-3 and ICD-10 classifications and included cancers of the (ICD-10: C00-C80, excluding non-melanoma skin cancer). Incident CVD diagnoses included angina pectoris (ICD-10: I20), acute myocardial infarction (ICD-10: I21), other acute ischemic heart diseases (ICD-10: I24), chronic ischemic heart diseases (ICD-10: I25), atrial fibrillation (ICD-10: I48), other cardiac arrhythmias (ICD-10: I49), heart failure (ICD-10: I50), cerebrovascular diseases including stroke (ICD-10: I60–I69), atherosclerosis (ICD-10: I70), and other peripheral vascular diseases (ICD-10: I73). Incident T2D diagnoses were defined as ICD-10 E11.

Covariates in UK Biobank included age, sex, country (England, Scotland, Wales), socioeconomic position (Townsend index, categorized in quantiles), educational level (highest, intermediate, lowest, other), BMI classes (underweight, normal weight, overweight, obesity), moderate-to-vigorous physical activity (metabolic equivalents of task-hours per week; categorized in quantiles), diet (healthy diet score, ranging 0-7), smoking status (never, former, current), alcohol consumption (never, former, current), sedentary behavior (hours per day, categorized in quantiles), and previous cancer screening (yes, no). Missing values were coded as missing.

We used weighted Cox proportional hazard regression to estimate hazard ratios and corresponding 95% confidence intervals for the associations of individual aptamers with cardiovascular disease, type 2 diabetes, and cancer. Weights were constructed based on each individual’s probability to be included in the study to account for the pre-selection of disease cases (9). The calculation of inclusion probabilities followed the sequential selection procedure used in the design of the multi‑endpoint case‑cohort study within EPIC. First, the eligible population was defined by applying the study‑specific inclusion and exclusion criteria to the full EPIC cohort. Participants who died and/or developed cancer, and/or experienced a stroke during follow‑up were then assigned an inclusion probability equal to 1. For all remaining participants, inclusion probabilities were computed as

P(in subcohort) + [1 – P(in subcohort)] × P(case | not in subcohort)

where:

- P(in subcohort) was estimated in the eligible population using a logistic regression model adjusted for study center;
- P(case | not in subchort) was estimated in the eligible population after excluding participants selected in the subcohort and those who died or developed cancer or had a stroke during follow-up, using a logistic regression model adjusted for the incidence of type 2 diabetes and/or cardiovascular disease (modelled using two binary indicators).

To validate this weighting approach, we conducted simulation studies to verify that the weighted analyses yielded unbiased estimates of associations between standard risk factors (e.g. smoking history, alcohol consumption, BMI, country, sex) and the risks of death, cancer, cardiovascular disease, stroke, and type 2 diabetes. Specifically, we replicated the multi‑endpoint case‑cohort selection procedure 100 times, recomputed inclusion probabilities and corresponding weights at each iteration, and confirmed that point estimates from the weighted analyses closely matched those obtained from unweighted analyses of the full eligible population.

**References**

1. Sudlow C, Gallacher J, Allen N, Beral V, Burton P, Danesh J, et al. UK Biobank: An Open Access Resource for Identifying the Causes of a Wide Range of Complex Diseases of Middle and Old Age. PLoS Med. 2015;12(3):e1001779.

2. Sun BB, Chiou J, Traylor M, Benner C, Hsu Y-H, Richardson TG, et al. Plasma proteomic associations with genetics and health in the UK Biobank. Nature. 2023;622(7982):329–38.

3. Eldjarn GH, Ferkingstad E, Lund SH, Helgason H, Magnusson OT, Gunnarsdottir K, et al. Large-scale plasma proteomics comparisons through genetics and disease associations. Nature. 2023;622(7982):348–58.

4. Wik L, Nordberg N, Broberg J, Björkesten J, Assarsson E, Henriksson S, et al. Proximity Extension Assay in Combination with Next-Generation Sequencing for High-throughput Proteome-wide Analysis. Mol Cell Proteomics. 2021;20.

5. Papier K, Atkins JR, Tong TYN, Gaitskell K, Desai T, Ogamba CF, et al. Identifying proteomic risk factors for cancer using prospective and exome analyses of 1463 circulating proteins and risk of 19 cancers in the UK Biobank. Nat Commun. 2024;15(1):4010.

6. Albanes D, Alcala K, Alcala N, Amos CI, Arslan AA, Bassett JK, et al. The blood proteome of imminent lung cancer diagnosis. Nat Commun. 2023;14(1):3042.

7. Trehearne A. Genetics, lifestyle and environment. Bundesgesundheitsblatt - Gesundheitsforschung - Gesundheitsschutz. 2016;59(3):361–7.

8. UK Biobank. Data providers and dates of data availability [Internet]. [cited 2025 Feb 12]. Available from: <https://biobank.ndph.ox.ac.uk/showcase/exinfo.cgi?src=Data_providers_and_dates>.

9. Binder DA. Fitting Cox's Proportional Hazards Models from Survey Data. Biometrika. 1992;79(1):139–47.

# Flow chart
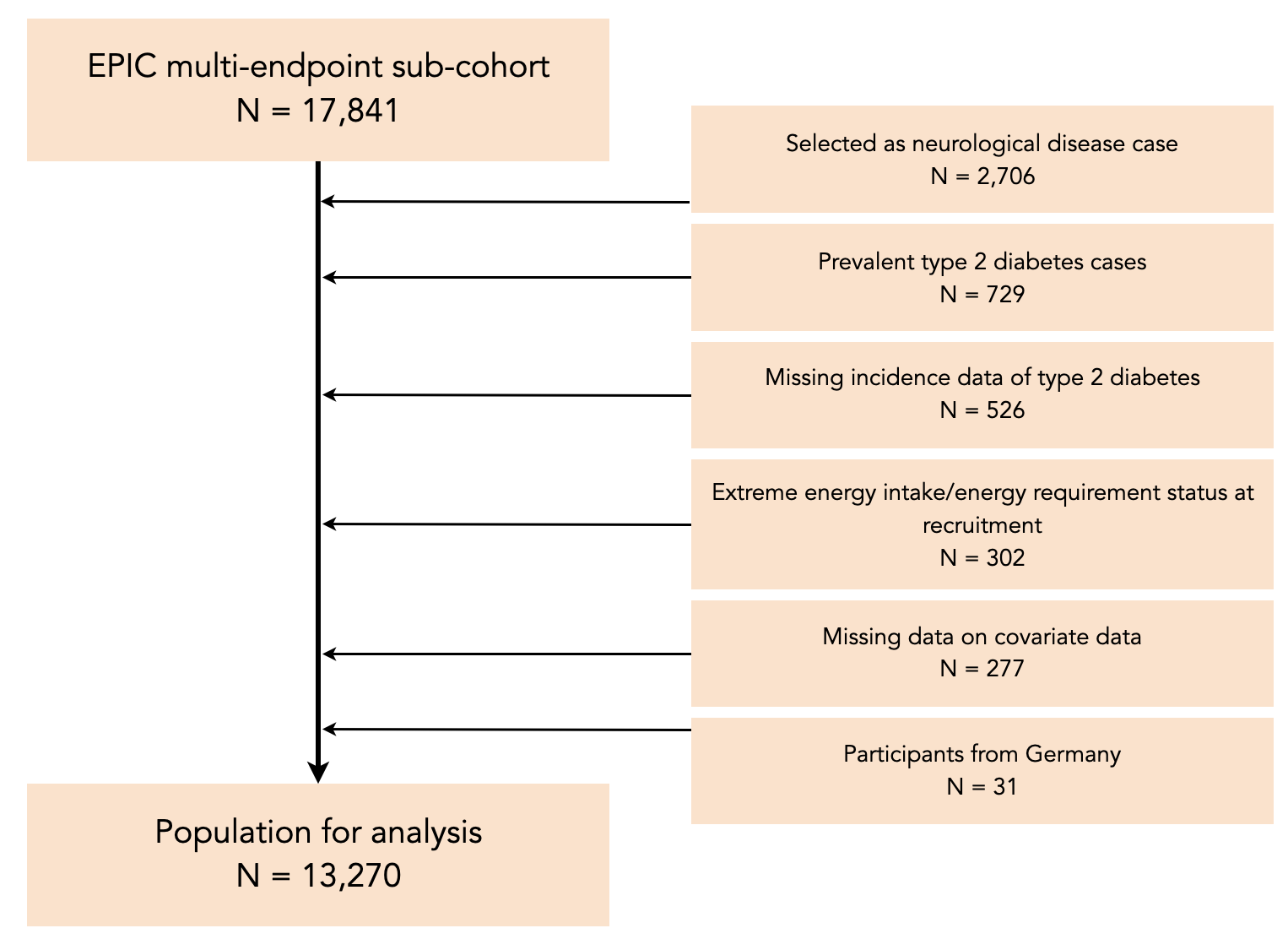
 of participant exclusions

# Pairwise correlation of multimorbidity-associated aptamers


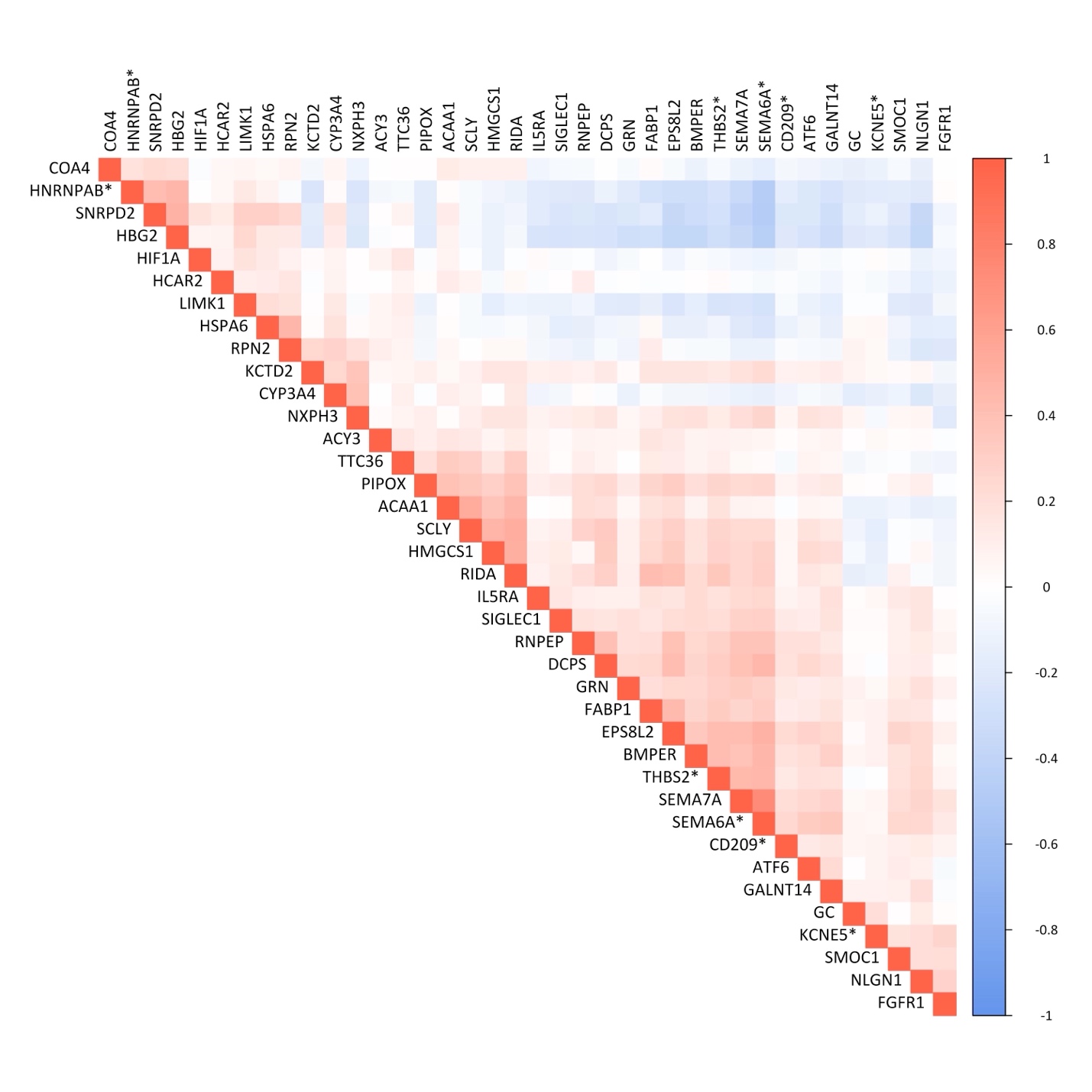


#
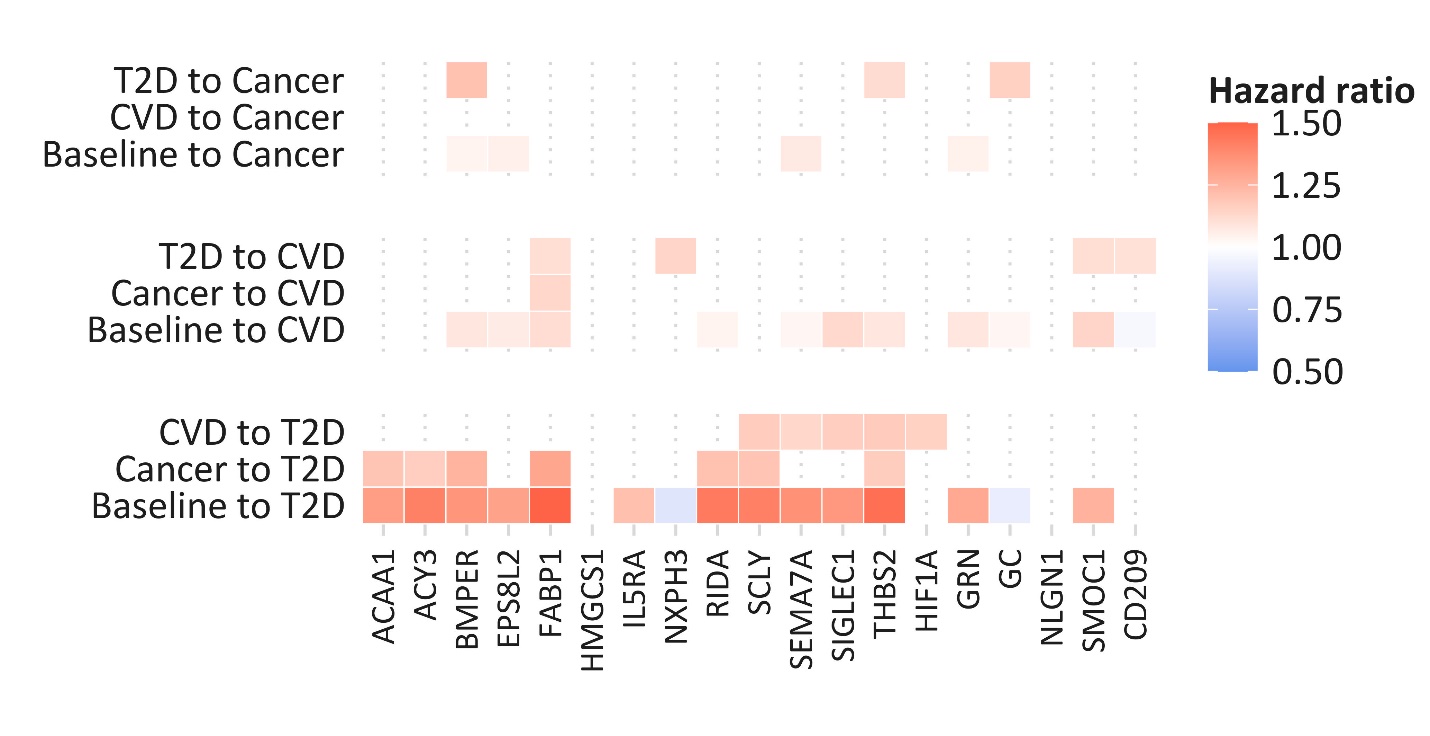
Cross-platform validation in UK Biobank

CVD. Cardiovascular disease; T2D: type 2 diabetes.

# Cox regression with adjustment for time since first disease


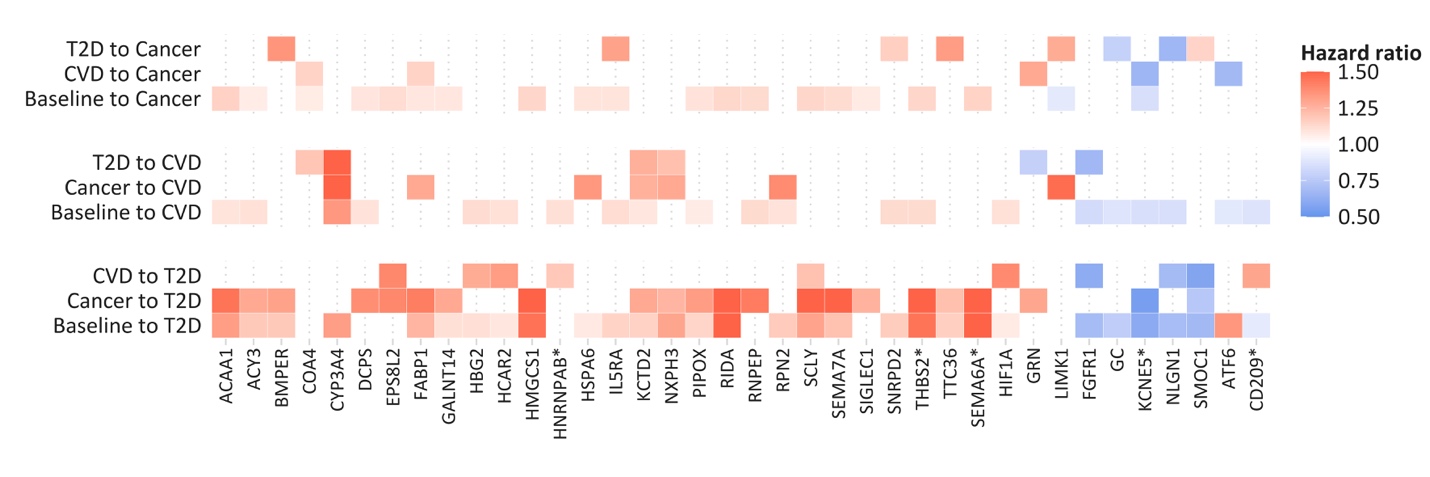


CVD. Cardiovascular disease; T2D: type 2 diabetes.

## Mutual adjustment models of multimorbidity-associated aptamers

| **Protein** | **Baseline to cancer** | **Baseline to T2D** | **Cancer to T2D** |
| --- | --- | --- | --- |
| HMGCS1 | 1.03 (95% CI: 0.95, 1.13) | 1.06 (95% CI: 0.95, 1.19) | 1.22 (95% CI: 0.87, 1.72) |
| RIDA | 1.00 (95% CI: 0.91, 1.10) | 1.68 (95% CI: 1.49, 1.88) | 1.17 (95% CI: 0.70, 1.95) |
| ACAA1 | 1.09 (95% CI: 1.01, 1.18) | 1.05 (95% CI: 0.96, 1.15) | 1.07 (95% CI: 0.73, 1.58) |
| SCLY | 1.07 (95% CI: 0.99, 1.16) | 0.96 (95% CI: 0.85, 1.07) | 1.56 (95% CI: 1.04, 2.33) |
| SEMA7A | 1.08 (95% CI: 0.98, 1.19) | 0.86 (95% CI: 0.74, 0.99) | 1.03 (95% CI: 0.68, 1.56) |
| SEMA6A* | 1.07 (95% CI: 0.95, 1.20) | 1.52 (95% CI: 1.27, 1.83) | 1.67 (95% CI: 0.81, 3.43) |
| CVD: cardiovascular disease; T2D: type 2 diabetes. | | | |

## Cross-platform confirmation with proportion of consistent effect directions and statistical significance in UK Biobank

| **Transition** | **Consistent direction of effect** | **FDR < 0.05 in UK Biobank** |
| --- | --- | --- |
| Baseline to CVD | 52.6% | 42.1% |
| Baseline to Cancer | 84.2% | 15.8% |
| Baseline to T2D | 78.9% | 78.9% |
| CVD to Cancer | 47.4% | 0.0% |
| CVD to T2D | 52.6% | 5.3% |
| Cancer to CVD | 57.9% | 5.3% |
| Cancer to T2D | 73.7% | 10.5% |
| T2D to CVD | 57.9% | 5.3% |
| T2D to Cancer | 47.4% | 0.0% |
| CVD: cardiovascular disease; FDR: false discovery rate; T2D: type 2 diabetes | | |

## Cox regression with first 1-5 years of follow-up excluded

| **Protein** | **Transition** | **1 year** | **2 years** | **3 years** | **4 years** | **5 years** |
| --- | --- | --- | --- | --- | --- | --- |
| ATF6 | Baseline to Cancer | 0.96 (95% CI: 0.89, 1.04) | 0.97 (95% CI: 0.90, 1.05) | 0.97 (95% CI: 0.89, 1.05) | 0.95 (95% CI: 0.88, 1.03) | 0.94 (95% CI: 0.86, 1.02) |
|  | CVD to Cancer | 0.70 (95% CI: 0.57, 0.85) | 0.69 (95% CI: 0.56, 0.85) | 0.69 (95% CI: 0.56, 0.86) | 0.69 (95% CI: 0.56, 0.85) | 0.68 (95% CI: 0.54, 0.85) |
|  | T2D to Cancer | 0.97 (95% CI: 0.77, 1.22) | 0.97 (95% CI: 0.77, 1.22) | 0.97 (95% CI: 0.77, 1.22) | 0.98 (95% CI: 0.78, 1.22) | 1.02 (95% CI: 0.82, 1.27) |
|  | Baseline to CVD | 0.90 (95% CI: 0.82, 0.98) | 0.90 (95% CI: 0.82, 0.98) | 0.90 (95% CI: 0.82, 0.99) | 0.92 (95% CI: 0.83, 1.01) | 0.92 (95% CI: 0.83, 1.01) |
|  | Cancer to CVD | 1.14 (95% CI: 0.87, 1.50) | 1.14 (95% CI: 0.87, 1.51) | 1.13 (95% CI: 0.85, 1.49) | 1.15 (95% CI: 0.87, 1.52) | 1.13 (95% CI: 0.84, 1.52) |
|  | T2D to CVD | 0.86 (95% CI: 0.67, 1.11) | 0.87 (95% CI: 0.67, 1.12) | 0.88 (95% CI: 0.68, 1.13) | 0.89 (95% CI: 0.69, 1.15) | 0.84 (95% CI: 0.66, 1.06) |
|  | Baseline to T2D | 1.36 (95% CI: 1.23, 1.51) | 1.35 (95% CI: 1.22, 1.50) | 1.34 (95% CI: 1.20, 1.49) | 1.31 (95% CI: 1.18, 1.46) | 1.28 (95% CI: 1.14, 1.44) |
|  | Cancer to T2D | 1.27 (95% CI: 0.92, 1.75) | 1.26 (95% CI: 0.91, 1.76) | 1.22 (95% CI: 0.87, 1.70) | 1.20 (95% CI: 0.86, 1.69) | 1.35 (95% CI: 0.98, 1.86) |
|  | CVD to T2D | 1.08 (95% CI: 0.77, 1.52) | 1.06 (95% CI: 0.75, 1.50) | 1.06 (95% CI: 0.75, 1.51) | 1.03 (95% CI: 0.71, 1.49) | 0.98 (95% CI: 0.68, 1.43) |
| FABP1 | Baseline to Cancer | 1.12 (95% CI: 1.05, 1.19) | 1.11 (95% CI: 1.05, 1.18) | 1.11 (95% CI: 1.04, 1.18) | 1.11 (95% CI: 1.04, 1.18) | 1.10 (95% CI: 1.03, 1.17) |
|  | CVD to Cancer | 1.14 (95% CI: 1.04, 1.25) | 1.14 (95% CI: 1.03, 1.25) | 1.13 (95% CI: 1.03, 1.25) | 1.13 (95% CI: 1.03, 1.25) | 1.16 (95% CI: 1.06, 1.28) |
|  | T2D to Cancer | 0.92 (95% CI: 0.72, 1.18) | 0.93 (95% CI: 0.73, 1.19) | 0.95 (95% CI: 0.74, 1.21) | 0.95 (95% CI: 0.75, 1.22) | 0.93 (95% CI: 0.72, 1.20) |
|  | Baseline to CVD | 1.07 (95% CI: 0.98, 1.16) | 1.08 (95% CI: 1.00, 1.18) | 1.07 (95% CI: 0.99, 1.17) | 1.09 (95% CI: 1.00, 1.18) | 1.07 (95% CI: 0.98, 1.17) |
|  | Cancer to CVD | 1.22 (95% CI: 1.00, 1.49) | 1.22 (95% CI: 1.00, 1.49) | 1.20 (95% CI: 0.98, 1.46) | 1.19 (95% CI: 0.97, 1.45) | 1.20 (95% CI: 0.97, 1.47) |
|  | T2D to CVD | 1.14 (95% CI: 0.93, 1.41) | 1.14 (95% CI: 0.93, 1.41) | 1.16 (95% CI: 0.93, 1.43) | 1.17 (95% CI: 0.94, 1.45) | 1.14 (95% CI: 0.91, 1.43) |
|  | Baseline to T2D | 1.30 (95% CI: 1.21, 1.40) | 1.31 (95% CI: 1.21, 1.41) | 1.32 (95% CI: 1.22, 1.43) | 1.29 (95% CI: 1.19, 1.40) | 1.30 (95% CI: 1.19, 1.41) |
|  | Cancer to T2D | 1.50 (95% CI: 1.23, 1.84) | 1.49 (95% CI: 1.21, 1.83) | 1.64 (95% CI: 1.34, 2.02) | 1.61 (95% CI: 1.31, 1.99) | 1.58 (95% CI: 1.29, 1.95) |
|  | CVD to T2D | 1.05 (95% CI: 0.86, 1.28) | 1.07 (95% CI: 0.88, 1.30) | 1.04 (95% CI: 0.84, 1.29) | 0.98 (95% CI: 0.73, 1.31) | 1.00 (95% CI: 0.77, 1.31) |
| RNPEP | Baseline to Cancer | 1.16 (95% CI: 1.07, 1.27) | 1.18 (95% CI: 1.08, 1.28) | 1.20 (95% CI: 1.10, 1.30) | 1.20 (95% CI: 1.10, 1.31) | 1.22 (95% CI: 1.11, 1.33) |
|  | CVD to Cancer | 1.03 (95% CI: 0.85, 1.25) | 1.02 (95% CI: 0.84, 1.23) | 1.03 (95% CI: 0.84, 1.25) | 1.05 (95% CI: 0.86, 1.27) | 1.05 (95% CI: 0.87, 1.27) |
|  | T2D to Cancer | 1.02 (95% CI: 0.83, 1.26) | 1.02 (95% CI: 0.83, 1.25) | 1.03 (95% CI: 0.84, 1.27) | 1.03 (95% CI: 0.83, 1.26) | 1.05 (95% CI: 0.84, 1.30) |
|  | Baseline to CVD | 1.12 (95% CI: 1.03, 1.23) | 1.11 (95% CI: 1.02, 1.22) | 1.11 (95% CI: 1.01, 1.22) | 1.14 (95% CI: 1.03, 1.26) | 1.16 (95% CI: 1.05, 1.29) |
|  | Cancer to CVD | 1.06 (95% CI: 0.80, 1.40) | 1.05 (95% CI: 0.79, 1.38) | 1.01 (95% CI: 0.76, 1.33) | 0.99 (95% CI: 0.74, 1.32) | 0.99 (95% CI: 0.74, 1.32) |
|  | T2D to CVD | 0.87 (95% CI: 0.69, 1.10) | 0.87 (95% CI: 0.69, 1.09) | 0.87 (95% CI: 0.69, 1.09) | 0.83 (95% CI: 0.67, 1.04) | 0.81 (95% CI: 0.65, 1.01) |
|  | Baseline to T2D | 1.07 (95% CI: 0.97, 1.18) | 1.06 (95% CI: 0.96, 1.18) | 1.06 (95% CI: 0.95, 1.18) | 1.06 (95% CI: 0.95, 1.18) | 1.06 (95% CI: 0.95, 1.19) |
|  | Cancer to T2D | 1.38 (95% CI: 1.16, 1.65) | 1.39 (95% CI: 1.16, 1.66) | 1.41 (95% CI: 1.18, 1.68) | 1.41 (95% CI: 1.18, 1.69) | 1.45 (95% CI: 1.22, 1.73) |
|  | CVD to T2D | 1.20 (95% CI: 0.92, 1.57) | 1.19 (95% CI: 0.90, 1.58) | 1.19 (95% CI: 0.90, 1.57) | 1.19 (95% CI: 0.89, 1.59) | 1.17 (95% CI: 0.87, 1.58) |
| HIF1A | Baseline to Cancer | 1.06 (95% CI: 0.99, 1.14) | 1.06 (95% CI: 0.99, 1.14) | 1.07 (95% CI: 1.00, 1.15) | 1.08 (95% CI: 1.00, 1.16) | 1.08 (95% CI: 1.00, 1.17) |
|  | CVD to Cancer | 1.04 (95% CI: 0.84, 1.29) | 1.04 (95% CI: 0.84, 1.30) | 1.05 (95% CI: 0.85, 1.31) | 1.06 (95% CI: 0.85, 1.32) | 1.06 (95% CI: 0.85, 1.33) |
|  | T2D to Cancer | 1.04 (95% CI: 0.85, 1.28) | 1.04 (95% CI: 0.85, 1.28) | 1.05 (95% CI: 0.85, 1.29) | 1.04 (95% CI: 0.85, 1.28) | 1.07 (95% CI: 0.87, 1.30) |
|  | Baseline to CVD | 1.11 (95% CI: 1.04, 1.18) | 1.12 (95% CI: 1.05, 1.19) | 1.13 (95% CI: 1.05, 1.20) | 1.12 (95% CI: 1.05, 1.20) | 1.13 (95% CI: 1.05, 1.21) |
|  | Cancer to CVD | 0.95 (95% CI: 0.76, 1.18) | 0.94 (95% CI: 0.76, 1.17) | 0.91 (95% CI: 0.72, 1.14) | 0.92 (95% CI: 0.74, 1.14) | 0.91 (95% CI: 0.73, 1.12) |
|  | T2D to CVD | 0.88 (95% CI: 0.70, 1.11) | 0.88 (95% CI: 0.70, 1.12) | 0.89 (95% CI: 0.70, 1.12) | 0.90 (95% CI: 0.72, 1.13) | 0.94 (95% CI: 0.76, 1.16) |
|  | Baseline to T2D | 1.07 (95% CI: 1.00, 1.15) | 1.09 (95% CI: 1.01, 1.17) | 1.10 (95% CI: 1.02, 1.18) | 1.09 (95% CI: 1.01, 1.18) | 1.11 (95% CI: 1.02, 1.20) |
|  | Cancer to T2D | 0.80 (95% CI: 0.58, 1.09) | 0.80 (95% CI: 0.59, 1.10) | 0.81 (95% CI: 0.60, 1.10) | 0.81 (95% CI: 0.58, 1.12) | 0.73 (95% CI: 0.53, 1.00) |
|  | CVD to T2D | 1.48 (95% CI: 1.18, 1.87) | 1.48 (95% CI: 1.19, 1.84) | 1.48 (95% CI: 1.21, 1.81) | 1.49 (95% CI: 1.23, 1.80) | 1.44 (95% CI: 1.21, 1.73) |
| SMOC1 | Baseline to Cancer | 1.00 (95% CI: 0.93, 1.08) | 1.00 (95% CI: 0.92, 1.07) | 1.01 (95% CI: 0.94, 1.09) | 1.00 (95% CI: 0.92, 1.08) | 1.01 (95% CI: 0.93, 1.09) |
|  | CVD to Cancer | 0.92 (95% CI: 0.75, 1.13) | 0.92 (95% CI: 0.75, 1.14) | 0.93 (95% CI: 0.75, 1.14) | 0.91 (95% CI: 0.73, 1.13) | 0.96 (95% CI: 0.78, 1.18) |
|  | T2D to Cancer | 1.15 (95% CI: 1.08, 1.23) | 1.15 (95% CI: 1.08, 1.23) | 1.15 (95% CI: 1.08, 1.23) | 1.15 (95% CI: 1.08, 1.23) | 1.14 (95% CI: 1.07, 1.22) |
|  | Baseline to CVD | 0.98 (95% CI: 0.89, 1.08) | 1.00 (95% CI: 0.91, 1.10) | 0.99 (95% CI: 0.89, 1.09) | 1.01 (95% CI: 0.91, 1.12) | 0.99 (95% CI: 0.89, 1.11) |
|  | Cancer to CVD | 0.84 (95% CI: 0.65, 1.10) | 0.87 (95% CI: 0.68, 1.13) | 0.84 (95% CI: 0.65, 1.09) | 0.82 (95% CI: 0.63, 1.07) | 0.86 (95% CI: 0.67, 1.11) |
|  | T2D to CVD | 0.87 (95% CI: 0.62, 1.22) | 0.88 (95% CI: 0.63, 1.22) | 0.88 (95% CI: 0.64, 1.22) | 0.90 (95% CI: 0.65, 1.24) | 0.91 (95% CI: 0.66, 1.24) |
|  | Baseline to T2D | 0.70 (95% CI: 0.61, 0.80) | 0.69 (95% CI: 0.60, 0.80) | 0.70 (95% CI: 0.61, 0.81) | 0.72 (95% CI: 0.62, 0.83) | 0.73 (95% CI: 0.63, 0.85) |
|  | Cancer to T2D | 0.74 (95% CI: 0.55, 1.00) | 0.75 (95% CI: 0.55, 1.01) | 0.74 (95% CI: 0.54, 1.01) | 0.77 (95% CI: 0.57, 1.04) | 0.75 (95% CI: 0.55, 1.02) |
|  | CVD to T2D | 0.61 (95% CI: 0.34, 1.08) | 0.62 (95% CI: 0.35, 1.08) | 0.62 (95% CI: 0.35, 1.09) | 0.67 (95% CI: 0.39, 1.14) | 0.65 (95% CI: 0.38, 1.11) |
| HCAR2 | Baseline to Cancer | 1.02 (95% CI: 0.96, 1.09) | 1.02 (95% CI: 0.95, 1.09) | 1.02 (95% CI: 0.96, 1.10) | 1.01 (95% CI: 0.94, 1.09) | 1.00 (95% CI: 0.92, 1.09) |
|  | CVD to Cancer | 1.15 (95% CI: 0.95, 1.38) | 1.15 (95% CI: 0.96, 1.38) | 1.15 (95% CI: 0.96, 1.38) | 1.18 (95% CI: 1.00, 1.40) | 1.19 (95% CI: 1.00, 1.41) |
|  | T2D to Cancer | 0.95 (95% CI: 0.73, 1.24) | 0.96 (95% CI: 0.74, 1.24) | 0.97 (95% CI: 0.74, 1.26) | 0.97 (95% CI: 0.75, 1.26) | 0.96 (95% CI: 0.74, 1.25) |
|  | Baseline to CVD | 1.11 (95% CI: 1.03, 1.19) | 1.11 (95% CI: 1.03, 1.20) | 1.11 (95% CI: 1.03, 1.20) | 1.13 (95% CI: 1.05, 1.23) | 1.12 (95% CI: 1.02, 1.22) |
|  | Cancer to CVD | 0.97 (95% CI: 0.73, 1.28) | 0.94 (95% CI: 0.70, 1.26) | 0.94 (95% CI: 0.71, 1.26) | 0.96 (95% CI: 0.71, 1.29) | 0.96 (95% CI: 0.71, 1.30) |
|  | T2D to CVD | 0.90 (95% CI: 0.66, 1.22) | 0.91 (95% CI: 0.67, 1.23) | 0.91 (95% CI: 0.68, 1.23) | 0.96 (95% CI: 0.73, 1.28) | 1.02 (95% CI: 0.79, 1.31) |
|  | Baseline to T2D | 1.06 (95% CI: 0.98, 1.15) | 1.06 (95% CI: 0.97, 1.15) | 1.06 (95% CI: 0.97, 1.15) | 1.06 (95% CI: 0.97, 1.15) | 1.04 (95% CI: 0.94, 1.14) |
|  | Cancer to T2D | 1.07 (95% CI: 0.85, 1.34) | 1.07 (95% CI: 0.85, 1.34) | 1.06 (95% CI: 0.83, 1.35) | 1.07 (95% CI: 0.85, 1.36) | 1.05 (95% CI: 0.81, 1.36) |
|  | CVD to T2D | 1.28 (95% CI: 1.02, 1.62) | 1.27 (95% CI: 1.00, 1.61) | 1.30 (95% CI: 1.03, 1.62) | 1.33 (95% CI: 1.08, 1.63) | 1.95 (95% CI: 1.58, 2.40) |
| HMGCS1 | Baseline to Cancer | 1.17 (95% CI: 1.07, 1.27) | 1.19 (95% CI: 1.09, 1.30) | 1.16 (95% CI: 1.06, 1.25) | 1.16 (95% CI: 1.06, 1.26) | 1.15 (95% CI: 1.06, 1.26) |
|  | CVD to Cancer | 1.08 (95% CI: 0.87, 1.35) | 1.09 (95% CI: 0.87, 1.37) | 1.09 (95% CI: 0.87, 1.38) | 1.08 (95% CI: 0.86, 1.37) | 1.09 (95% CI: 0.85, 1.38) |
|  | T2D to Cancer | 1.01 (95% CI: 0.82, 1.25) | 1.02 (95% CI: 0.82, 1.25) | 1.02 (95% CI: 0.83, 1.25) | 1.03 (95% CI: 0.83, 1.27) | 1.00 (95% CI: 0.81, 1.24) |
|  | Baseline to CVD | 0.96 (95% CI: 0.88, 1.05) | 0.96 (95% CI: 0.87, 1.05) | 0.95 (95% CI: 0.86, 1.04) | 0.97 (95% CI: 0.88, 1.07) | 0.97 (95% CI: 0.88, 1.07) |
|  | Cancer to CVD | 1.18 (95% CI: 0.90, 1.54) | 1.18 (95% CI: 0.90, 1.55) | 1.17 (95% CI: 0.90, 1.53) | 1.18 (95% CI: 0.91, 1.54) | 1.17 (95% CI: 0.90, 1.53) |
|  | T2D to CVD | 0.99 (95% CI: 0.82, 1.21) | 0.99 (95% CI: 0.81, 1.20) | 0.97 (95% CI: 0.80, 1.18) | 0.95 (95% CI: 0.78, 1.15) | 0.91 (95% CI: 0.75, 1.09) |
|  | Baseline to T2D | 1.51 (95% CI: 1.37, 1.66) | 1.51 (95% CI: 1.36, 1.67) | 1.50 (95% CI: 1.35, 1.66) | 1.43 (95% CI: 1.29, 1.60) | 1.39 (95% CI: 1.24, 1.56) |
|  | Cancer to T2D | 2.23 (95% CI: 1.65, 3.00) | 2.23 (95% CI: 1.63, 3.05) | 2.49 (95% CI: 1.84, 3.37) | 2.39 (95% CI: 1.76, 3.25) | 2.61 (95% CI: 2.00, 3.41) |
|  | CVD to T2D | 1.17 (95% CI: 0.88, 1.55) | 1.17 (95% CI: 0.88, 1.55) | 1.15 (95% CI: 0.86, 1.53) | 1.11 (95% CI: 0.84, 1.47) | 1.15 (95% CI: 0.87, 1.52) |
| HSPA6 | Baseline to Cancer | 1.09 (95% CI: 1.04, 1.14) | 1.09 (95% CI: 1.05, 1.15) | 1.09 (95% CI: 1.05, 1.15) | 1.10 (95% CI: 1.05, 1.15) | 1.11 (95% CI: 1.06, 1.16) |
|  | CVD to Cancer | 0.96 (95% CI: 0.77, 1.20) | 0.98 (95% CI: 0.78, 1.22) | 1.00 (95% CI: 0.80, 1.25) | 1.00 (95% CI: 0.80, 1.25) | 0.99 (95% CI: 0.78, 1.25) |
|  | T2D to Cancer | 1.03 (95% CI: 0.79, 1.36) | 1.04 (95% CI: 0.79, 1.36) | 1.04 (95% CI: 0.78, 1.37) | 1.03 (95% CI: 0.78, 1.37) | 1.01 (95% CI: 0.76, 1.35) |
|  | Baseline to CVD | 1.05 (95% CI: 0.98, 1.13) | 1.05 (95% CI: 0.98, 1.13) | 1.03 (95% CI: 0.95, 1.11) | 1.01 (95% CI: 0.93, 1.10) | 1.00 (95% CI: 0.92, 1.09) |
|  | Cancer to CVD | 1.30 (95% CI: 1.17, 1.45) | 1.29 (95% CI: 1.16, 1.44) | 1.28 (95% CI: 1.15, 1.42) | 1.30 (95% CI: 1.16, 1.46) | 1.34 (95% CI: 1.20, 1.51) |
|  | T2D to CVD | 1.19 (95% CI: 0.88, 1.61) | 1.19 (95% CI: 0.88, 1.61) | 1.19 (95% CI: 0.88, 1.61) | 1.32 (95% CI: 1.01, 1.73) | 1.40 (95% CI: 1.09, 1.81) |
|  | Baseline to T2D | 1.07 (95% CI: 1.00, 1.14) | 1.07 (95% CI: 1.00, 1.14) | 1.07 (95% CI: 1.00, 1.15) | 1.08 (95% CI: 1.01, 1.15) | 1.09 (95% CI: 1.01, 1.16) |
|  | Cancer to T2D | 0.90 (95% CI: 0.64, 1.27) | 0.89 (95% CI: 0.63, 1.26) | 0.88 (95% CI: 0.62, 1.26) | 0.91 (95% CI: 0.64, 1.29) | 0.89 (95% CI: 0.62, 1.29) |
|  | CVD to T2D | 0.95 (95% CI: 0.71, 1.26) | 0.94 (95% CI: 0.70, 1.24) | 0.98 (95% CI: 0.74, 1.29) | 0.88 (95% CI: 0.66, 1.17) | 0.88 (95% CI: 0.65, 1.18) |
| IL5RA | Baseline to Cancer | 1.10 (95% CI: 1.02, 1.19) | 1.09 (95% CI: 1.01, 1.17) | 1.09 (95% CI: 1.01, 1.18) | 1.09 (95% CI: 1.00, 1.18) | 1.10 (95% CI: 1.00, 1.20) |
|  | CVD to Cancer | 1.18 (95% CI: 1.01, 1.37) | 1.16 (95% CI: 0.99, 1.36) | 1.18 (95% CI: 1.02, 1.38) | 1.19 (95% CI: 1.03, 1.38) | 1.19 (95% CI: 1.01, 1.41) |
|  | T2D to Cancer | 1.32 (95% CI: 1.14, 1.53) | 1.32 (95% CI: 1.14, 1.52) | 1.33 (95% CI: 1.16, 1.53) | 1.32 (95% CI: 1.15, 1.52) | 1.36 (95% CI: 1.17, 1.58) |
|  | Baseline to CVD | 1.10 (95% CI: 1.01, 1.19) | 1.09 (95% CI: 1.00, 1.19) | 1.09 (95% CI: 1.00, 1.19) | 1.13 (95% CI: 1.03, 1.23) | 1.11 (95% CI: 1.01, 1.23) |
|  | Cancer to CVD | 0.85 (95% CI: 0.64, 1.12) | 0.82 (95% CI: 0.61, 1.08) | 0.79 (95% CI: 0.59, 1.05) | 0.79 (95% CI: 0.59, 1.06) | 0.81 (95% CI: 0.60, 1.10) |
|  | T2D to CVD | 1.11 (95% CI: 0.89, 1.38) | 1.10 (95% CI: 0.89, 1.37) | 1.09 (95% CI: 0.88, 1.35) | 1.04 (95% CI: 0.82, 1.31) | 1.05 (95% CI: 0.83, 1.34) |
|  | Baseline to T2D | 1.22 (95% CI: 1.12, 1.33) | 1.21 (95% CI: 1.10, 1.32) | 1.20 (95% CI: 1.10, 1.31) | 1.19 (95% CI: 1.08, 1.31) | 1.19 (95% CI: 1.07, 1.31) |
|  | Cancer to T2D | 1.24 (95% CI: 1.00, 1.54) | 1.25 (95% CI: 1.01, 1.55) | 1.24 (95% CI: 0.99, 1.56) | 1.26 (95% CI: 1.01, 1.58) | 1.27 (95% CI: 1.01, 1.59) |
|  | CVD to T2D | 1.06 (95% CI: 0.80, 1.41) | 1.06 (95% CI: 0.80, 1.40) | 1.04 (95% CI: 0.78, 1.38) | 1.03 (95% CI: 0.78, 1.37) | 1.00 (95% CI: 0.74, 1.34) |
| RIDA | Baseline to Cancer | 1.15 (95% CI: 1.05, 1.25) | 1.17 (95% CI: 1.07, 1.27) | 1.16 (95% CI: 1.07, 1.27) | 1.16 (95% CI: 1.06, 1.26) | 1.15 (95% CI: 1.05, 1.25) |
|  | CVD to Cancer | 1.20 (95% CI: 0.99, 1.45) | 1.21 (95% CI: 0.99, 1.47) | 1.23 (95% CI: 1.01, 1.49) | 1.23 (95% CI: 1.01, 1.50) | 1.22 (95% CI: 0.99, 1.50) |
|  | T2D to Cancer | 1.20 (95% CI: 0.99, 1.46) | 1.20 (95% CI: 0.99, 1.47) | 1.22 (95% CI: 1.01, 1.49) | 1.23 (95% CI: 1.01, 1.50) | 1.20 (95% CI: 0.98, 1.46) |
|  | Baseline to CVD | 1.10 (95% CI: 1.00, 1.20) | 1.09 (95% CI: 1.00, 1.19) | 1.08 (95% CI: 0.98, 1.18) | 1.09 (95% CI: 0.99, 1.20) | 1.06 (95% CI: 0.96, 1.17) |
|  | Cancer to CVD | 1.09 (95% CI: 0.87, 1.36) | 1.09 (95% CI: 0.87, 1.37) | 1.09 (95% CI: 0.87, 1.36) | 1.11 (95% CI: 0.89, 1.40) | 1.09 (95% CI: 0.87, 1.37) |
|  | T2D to CVD | 1.01 (95% CI: 0.79, 1.28) | 1.01 (95% CI: 0.79, 1.28) | 1.01 (95% CI: 0.79, 1.27) | 1.03 (95% CI: 0.81, 1.31) | 0.98 (95% CI: 0.77, 1.24) |
|  | Baseline to T2D | 1.77 (95% CI: 1.63, 1.92) | 1.78 (95% CI: 1.64, 1.94) | 1.80 (95% CI: 1.65, 1.96) | 1.75 (95% CI: 1.60, 1.91) | 1.74 (95% CI: 1.58, 1.91) |
|  | Cancer to T2D | 2.19 (95% CI: 1.71, 2.80) | 2.19 (95% CI: 1.71, 2.81) | 2.24 (95% CI: 1.74, 2.90) | 2.21 (95% CI: 1.70, 2.86) | 2.13 (95% CI: 1.62, 2.79) |
|  | CVD to T2D | 1.26 (95% CI: 0.94, 1.68) | 1.26 (95% CI: 0.94, 1.68) | 1.21 (95% CI: 0.90, 1.62) | 1.21 (95% CI: 0.89, 1.65) | 1.24 (95% CI: 0.91, 1.68) |
| BMPER | Baseline to Cancer | 1.03 (95% CI: 0.96, 1.11) | 1.04 (95% CI: 0.97, 1.12) | 1.06 (95% CI: 0.98, 1.14) | 1.06 (95% CI: 0.98, 1.15) | 1.05 (95% CI: 0.97, 1.14) |
|  | CVD to Cancer | 1.03 (95% CI: 0.86, 1.23) | 1.03 (95% CI: 0.86, 1.24) | 1.03 (95% CI: 0.86, 1.24) | 1.03 (95% CI: 0.87, 1.23) | 1.02 (95% CI: 0.84, 1.24) |
|  | T2D to Cancer | 1.28 (95% CI: 1.09, 1.50) | 1.27 (95% CI: 1.08, 1.49) | 1.26 (95% CI: 1.08, 1.47) | 1.24 (95% CI: 1.07, 1.44) | 1.22 (95% CI: 1.06, 1.41) |
|  | Baseline to CVD | 1.03 (95% CI: 0.95, 1.12) | 1.04 (95% CI: 0.95, 1.13) | 1.06 (95% CI: 0.97, 1.16) | 1.08 (95% CI: 0.98, 1.18) | 1.08 (95% CI: 0.99, 1.19) |
|  | Cancer to CVD | 0.94 (95% CI: 0.76, 1.17) | 0.94 (95% CI: 0.74, 1.18) | 0.92 (95% CI: 0.73, 1.17) | 0.93 (95% CI: 0.74, 1.17) | 0.91 (95% CI: 0.72, 1.16) |
|  | T2D to CVD | 1.07 (95% CI: 0.79, 1.45) | 1.07 (95% CI: 0.79, 1.46) | 1.08 (95% CI: 0.80, 1.45) | 1.06 (95% CI: 0.78, 1.45) | 1.07 (95% CI: 0.78, 1.47) |
|  | Baseline to T2D | 1.18 (95% CI: 1.09, 1.28) | 1.18 (95% CI: 1.08, 1.27) | 1.16 (95% CI: 1.06, 1.26) | 1.15 (95% CI: 1.06, 1.25) | 1.14 (95% CI: 1.04, 1.24) |
|  | Cancer to T2D | 1.29 (95% CI: 1.12, 1.49) | 1.29 (95% CI: 1.12, 1.48) | 1.26 (95% CI: 1.09, 1.46) | 1.23 (95% CI: 1.05, 1.43) | 1.21 (95% CI: 1.02, 1.45) |
|  | CVD to T2D | 1.06 (95% CI: 0.70, 1.59) | 1.06 (95% CI: 0.71, 1.60) | 1.05 (95% CI: 0.69, 1.61) | 1.05 (95% CI: 0.68, 1.62) | 0.97 (95% CI: 0.62, 1.51) |
| GC | Baseline to Cancer | 0.97 (95% CI: 0.89, 1.05) | 0.97 (95% CI: 0.89, 1.05) | 0.96 (95% CI: 0.88, 1.04) | 0.96 (95% CI: 0.88, 1.05) | 0.97 (95% CI: 0.88, 1.06) |
|  | CVD to Cancer | 0.86 (95% CI: 0.72, 1.04) | 0.86 (95% CI: 0.71, 1.04) | 0.87 (95% CI: 0.71, 1.05) | 0.86 (95% CI: 0.71, 1.04) | 0.84 (95% CI: 0.70, 1.01) |
|  | T2D to Cancer | 0.83 (95% CI: 0.72, 0.95) | 0.83 (95% CI: 0.73, 0.95) | 0.83 (95% CI: 0.73, 0.96) | 0.84 (95% CI: 0.73, 0.96) | 0.83 (95% CI: 0.73, 0.94) |
|  | Baseline to CVD | 0.91 (95% CI: 0.85, 0.97) | 0.90 (95% CI: 0.85, 0.96) | 0.89 (95% CI: 0.83, 0.95) | 0.88 (95% CI: 0.83, 0.95) | 0.89 (95% CI: 0.83, 0.95) |
|  | Cancer to CVD | 1.26 (95% CI: 0.94, 1.70) | 1.26 (95% CI: 0.94, 1.69) | 1.28 (95% CI: 0.95, 1.72) | 1.30 (95% CI: 0.96, 1.76) | 1.28 (95% CI: 0.94, 1.74) |
|  | T2D to CVD | 1.01 (95% CI: 0.79, 1.28) | 1.02 (95% CI: 0.80, 1.29) | 1.02 (95% CI: 0.81, 1.29) | 1.02 (95% CI: 0.81, 1.29) | 1.01 (95% CI: 0.80, 1.29) |
|  | Baseline to T2D | 0.80 (95% CI: 0.76, 0.85) | 0.80 (95% CI: 0.76, 0.85) | 0.81 (95% CI: 0.76, 0.85) | 0.81 (95% CI: 0.77, 0.86) | 0.83 (95% CI: 0.78, 0.88) |
|  | Cancer to T2D | 0.92 (95% CI: 0.73, 1.16) | 0.91 (95% CI: 0.73, 1.15) | 0.90 (95% CI: 0.71, 1.14) | 0.88 (95% CI: 0.68, 1.14) | 0.87 (95% CI: 0.67, 1.11) |
|  | CVD to T2D | 1.04 (95% CI: 0.74, 1.47) | 1.06 (95% CI: 0.75, 1.49) | 1.06 (95% CI: 0.75, 1.50) | 0.98 (95% CI: 0.70, 1.36) | 0.97 (95% CI: 0.70, 1.34) |
| NLGN1 | Baseline to Cancer | 1.04 (95% CI: 0.98, 1.12) | 1.04 (95% CI: 0.97, 1.11) | 1.04 (95% CI: 0.97, 1.11) | 1.04 (95% CI: 0.97, 1.12) | 1.06 (95% CI: 0.98, 1.13) |
|  | CVD to Cancer | 0.85 (95% CI: 0.68, 1.08) | 0.83 (95% CI: 0.66, 1.06) | 0.82 (95% CI: 0.64, 1.04) | 0.82 (95% CI: 0.65, 1.04) | 0.83 (95% CI: 0.65, 1.05) |
|  | T2D to Cancer | 0.62 (95% CI: 0.47, 0.82) | 0.62 (95% CI: 0.47, 0.82) | 0.63 (95% CI: 0.47, 0.83) | 0.63 (95% CI: 0.48, 0.84) | 0.66 (95% CI: 0.50, 0.87) |
|  | Baseline to CVD | 0.86 (95% CI: 0.78, 0.96) | 0.86 (95% CI: 0.78, 0.96) | 0.89 (95% CI: 0.80, 0.99) | 0.91 (95% CI: 0.81, 1.01) | 0.90 (95% CI: 0.81, 1.00) |
|  | Cancer to CVD | 0.78 (95% CI: 0.57, 1.07) | 0.79 (95% CI: 0.58, 1.08) | 0.78 (95% CI: 0.57, 1.08) | 0.77 (95% CI: 0.55, 1.07) | 0.75 (95% CI: 0.52, 1.07) |
|  | T2D to CVD | 1.02 (95% CI: 0.60, 1.73) | 1.02 (95% CI: 0.60, 1.75) | 1.04 (95% CI: 0.61, 1.76) | 1.01 (95% CI: 0.55, 1.86) | 0.99 (95% CI: 0.51, 1.90) |
|  | Baseline to T2D | 0.73 (95% CI: 0.62, 0.86) | 0.73 (95% CI: 0.62, 0.86) | 0.72 (95% CI: 0.60, 0.85) | 0.73 (95% CI: 0.61, 0.87) | 0.72 (95% CI: 0.59, 0.87) |
|  | Cancer to T2D | 0.74 (95% CI: 0.51, 1.05) | 0.74 (95% CI: 0.52, 1.06) | 0.75 (95% CI: 0.53, 1.07) | 0.74 (95% CI: 0.52, 1.05) | 0.78 (95% CI: 0.54, 1.12) |
|  | CVD to T2D | 0.69 (95% CI: 0.48, 0.98) | 0.70 (95% CI: 0.49, 1.01) | 0.69 (95% CI: 0.48, 1.00) | 0.73 (95% CI: 0.53, 1.02) | 0.73 (95% CI: 0.53, 1.02) |
| ACAA1 | Baseline to Cancer | 1.17 (95% CI: 1.07, 1.27) | 1.18 (95% CI: 1.09, 1.29) | 1.17 (95% CI: 1.08, 1.27) | 1.18 (95% CI: 1.09, 1.28) | 1.18 (95% CI: 1.09, 1.28) |
|  | CVD to Cancer | 1.14 (95% CI: 0.94, 1.37) | 1.13 (95% CI: 0.94, 1.37) | 1.15 (95% CI: 0.95, 1.39) | 1.17 (95% CI: 0.97, 1.42) | 1.20 (95% CI: 0.99, 1.45) |
|  | T2D to Cancer | 1.01 (95% CI: 0.82, 1.24) | 1.00 (95% CI: 0.81, 1.23) | 1.01 (95% CI: 0.83, 1.24) | 1.02 (95% CI: 0.83, 1.25) | 1.02 (95% CI: 0.83, 1.26) |
|  | Baseline to CVD | 1.07 (95% CI: 0.99, 1.16) | 1.09 (95% CI: 1.00, 1.18) | 1.09 (95% CI: 1.01, 1.18) | 1.10 (95% CI: 1.02, 1.20) | 1.10 (95% CI: 1.01, 1.19) |
|  | Cancer to CVD | 1.12 (95% CI: 0.91, 1.37) | 1.11 (95% CI: 0.90, 1.38) | 1.10 (95% CI: 0.89, 1.36) | 1.09 (95% CI: 0.88, 1.36) | 1.06 (95% CI: 0.84, 1.32) |
|  | T2D to CVD | 1.04 (95% CI: 0.85, 1.26) | 1.03 (95% CI: 0.85, 1.25) | 1.04 (95% CI: 0.86, 1.26) | 1.01 (95% CI: 0.83, 1.23) | 1.01 (95% CI: 0.82, 1.24) |
|  | Baseline to T2D | 1.33 (95% CI: 1.23, 1.44) | 1.32 (95% CI: 1.22, 1.43) | 1.31 (95% CI: 1.21, 1.42) | 1.27 (95% CI: 1.16, 1.38) | 1.24 (95% CI: 1.14, 1.36) |
|  | Cancer to T2D | 1.44 (95% CI: 1.22, 1.71) | 1.44 (95% CI: 1.21, 1.70) | 1.46 (95% CI: 1.23, 1.74) | 1.46 (95% CI: 1.22, 1.74) | 1.41 (95% CI: 1.17, 1.71) |
|  | CVD to T2D | 1.28 (95% CI: 0.95, 1.72) | 1.28 (95% CI: 0.95, 1.72) | 1.30 (95% CI: 0.96, 1.75) | 1.18 (95% CI: 0.87, 1.62) | 1.15 (95% CI: 0.83, 1.58) |
| SNRPD2 | Baseline to Cancer | 1.04 (95% CI: 0.95, 1.14) | 1.04 (95% CI: 0.94, 1.14) | 1.05 (95% CI: 0.95, 1.15) | 1.00 (95% CI: 0.91, 1.11) | 1.00 (95% CI: 0.90, 1.12) |
|  | CVD to Cancer | 1.12 (95% CI: 0.93, 1.36) | 1.14 (95% CI: 0.94, 1.37) | 1.13 (95% CI: 0.94, 1.37) | 1.11 (95% CI: 0.92, 1.34) | 1.14 (95% CI: 0.95, 1.37) |
|  | T2D to Cancer | 1.20 (95% CI: 1.05, 1.38) | 1.20 (95% CI: 1.05, 1.37) | 1.19 (95% CI: 1.05, 1.36) | 1.18 (95% CI: 1.03, 1.34) | 1.18 (95% CI: 1.03, 1.36) |
|  | Baseline to CVD | 1.11 (95% CI: 1.03, 1.18) | 1.11 (95% CI: 1.03, 1.19) | 1.11 (95% CI: 1.03, 1.19) | 1.09 (95% CI: 1.00, 1.18) | 1.09 (95% CI: 1.00, 1.19) |
|  | Cancer to CVD | 1.07 (95% CI: 0.88, 1.30) | 1.08 (95% CI: 0.90, 1.30) | 1.09 (95% CI: 0.91, 1.31) | 1.11 (95% CI: 0.93, 1.31) | 1.11 (95% CI: 0.93, 1.32) |
|  | T2D to CVD | 1.02 (95% CI: 0.85, 1.23) | 1.03 (95% CI: 0.84, 1.26) | 1.03 (95% CI: 0.84, 1.26) | 1.07 (95% CI: 0.90, 1.27) | 1.09 (95% CI: 0.93, 1.27) |
|  | Baseline to T2D | 1.15 (95% CI: 1.07, 1.24) | 1.15 (95% CI: 1.06, 1.24) | 1.16 (95% CI: 1.07, 1.25) | 1.14 (95% CI: 1.05, 1.24) | 1.14 (95% CI: 1.04, 1.24) |
|  | Cancer to T2D | 1.01 (95% CI: 0.75, 1.37) | 1.02 (95% CI: 0.75, 1.38) | 1.03 (95% CI: 0.76, 1.40) | 1.04 (95% CI: 0.77, 1.39) | 0.99 (95% CI: 0.70, 1.39) |
|  | CVD to T2D | 1.01 (95% CI: 0.74, 1.38) | 1.01 (95% CI: 0.75, 1.38) | 1.05 (95% CI: 0.78, 1.41) | 1.02 (95% CI: 0.75, 1.40) | 1.00 (95% CI: 0.74, 1.37) |
| HBG2 | Baseline to Cancer | 1.01 (95% CI: 0.93, 1.10) | 1.00 (95% CI: 0.92, 1.09) | 1.02 (95% CI: 0.94, 1.11) | 1.03 (95% CI: 0.94, 1.12) | 1.02 (95% CI: 0.93, 1.13) |
|  | CVD to Cancer | 1.18 (95% CI: 0.99, 1.41) | 1.19 (95% CI: 1.00, 1.42) | 1.19 (95% CI: 1.00, 1.41) | 1.19 (95% CI: 0.99, 1.43) | 1.17 (95% CI: 0.98, 1.40) |
|  | T2D to Cancer | 1.25 (95% CI: 1.00, 1.56) | 1.25 (95% CI: 1.01, 1.55) | 1.24 (95% CI: 1.00, 1.55) | 1.24 (95% CI: 1.00, 1.55) | 1.23 (95% CI: 0.99, 1.53) |
|  | Baseline to CVD | 1.11 (95% CI: 1.02, 1.21) | 1.10 (95% CI: 1.01, 1.20) | 1.09 (95% CI: 1.00, 1.20) | 1.08 (95% CI: 0.97, 1.19) | 1.05 (95% CI: 0.94, 1.16) |
|  | Cancer to CVD | 1.22 (95% CI: 0.99, 1.52) | 1.22 (95% CI: 0.99, 1.50) | 1.22 (95% CI: 0.98, 1.52) | 1.22 (95% CI: 0.98, 1.52) | 1.24 (95% CI: 0.99, 1.57) |
|  | T2D to CVD | 0.98 (95% CI: 0.76, 1.27) | 0.97 (95% CI: 0.75, 1.26) | 0.97 (95% CI: 0.75, 1.26) | 1.02 (95% CI: 0.79, 1.30) | 1.00 (95% CI: 0.78, 1.29) |
|  | Baseline to T2D | 1.13 (95% CI: 1.02, 1.24) | 1.12 (95% CI: 1.02, 1.24) | 1.13 (95% CI: 1.03, 1.25) | 1.13 (95% CI: 1.03, 1.25) | 1.11 (95% CI: 1.00, 1.24) |
|  | Cancer to T2D | 0.93 (95% CI: 0.70, 1.24) | 0.93 (95% CI: 0.69, 1.24) | 0.94 (95% CI: 0.71, 1.26) | 0.96 (95% CI: 0.73, 1.26) | 0.92 (95% CI: 0.67, 1.25) |
|  | CVD to T2D | 1.36 (95% CI: 1.10, 1.69) | 1.35 (95% CI: 1.09, 1.67) | 1.36 (95% CI: 1.11, 1.66) | 1.37 (95% CI: 1.11, 1.68) | 1.36 (95% CI: 1.12, 1.66) |
| DCPS | Baseline to Cancer | 1.12 (95% CI: 1.06, 1.18) | 1.13 (95% CI: 1.07, 1.20) | 1.13 (95% CI: 1.07, 1.20) | 1.13 (95% CI: 1.06, 1.20) | 1.12 (95% CI: 1.06, 1.20) |
|  | CVD to Cancer | 1.08 (95% CI: 0.93, 1.27) | 1.08 (95% CI: 0.92, 1.26) | 1.09 (95% CI: 0.94, 1.27) | 1.09 (95% CI: 0.93, 1.28) | 0.97 (95% CI: 0.81, 1.16) |
|  | T2D to Cancer | 1.07 (95% CI: 0.91, 1.25) | 1.07 (95% CI: 0.91, 1.26) | 1.08 (95% CI: 0.92, 1.27) | 1.09 (95% CI: 0.93, 1.27) | 1.12 (95% CI: 0.97, 1.30) |
|  | Baseline to CVD | 1.13 (95% CI: 1.06, 1.20) | 1.13 (95% CI: 1.06, 1.21) | 1.13 (95% CI: 1.05, 1.21) | 1.14 (95% CI: 1.06, 1.23) | 1.15 (95% CI: 1.06, 1.24) |
|  | Cancer to CVD | 1.18 (95% CI: 1.00, 1.40) | 1.17 (95% CI: 0.99, 1.40) | 1.16 (95% CI: 0.97, 1.39) | 1.15 (95% CI: 0.96, 1.38) | 1.16 (95% CI: 0.92, 1.45) |
|  | T2D to CVD | 0.89 (95% CI: 0.69, 1.16) | 0.89 (95% CI: 0.69, 1.17) | 0.89 (95% CI: 0.69, 1.16) | 0.87 (95% CI: 0.67, 1.15) | 0.81 (95% CI: 0.63, 1.05) |
|  | Baseline to T2D | 1.03 (95% CI: 0.94, 1.13) | 1.02 (95% CI: 0.92, 1.12) | 1.01 (95% CI: 0.92, 1.12) | 0.99 (95% CI: 0.89, 1.10) | 0.96 (95% CI: 0.87, 1.06) |
|  | Cancer to T2D | 1.22 (95% CI: 1.01, 1.48) | 1.22 (95% CI: 0.99, 1.50) | 1.23 (95% CI: 0.99, 1.54) | 1.23 (95% CI: 0.94, 1.62) | 1.49 (95% CI: 1.15, 1.93) |
|  | CVD to T2D | 1.04 (95% CI: 0.80, 1.35) | 1.04 (95% CI: 0.80, 1.35) | 1.05 (95% CI: 0.80, 1.37) | 1.04 (95% CI: 0.79, 1.37) | 1.05 (95% CI: 0.80, 1.39) |
| COA4 | Baseline to Cancer | 1.05 (95% CI: 0.98, 1.13) | 1.05 (95% CI: 0.98, 1.13) | 1.06 (95% CI: 0.98, 1.14) | 1.07 (95% CI: 0.99, 1.15) | 1.07 (95% CI: 0.99, 1.15) |
|  | CVD to Cancer | 1.20 (95% CI: 1.11, 1.31) | 1.19 (95% CI: 1.09, 1.30) | 1.18 (95% CI: 1.08, 1.30) | 1.19 (95% CI: 1.09, 1.30) | 1.19 (95% CI: 1.09, 1.30) |
|  | T2D to Cancer | 1.05 (95% CI: 0.91, 1.21) | 1.05 (95% CI: 0.91, 1.20) | 1.05 (95% CI: 0.91, 1.20) | 1.04 (95% CI: 0.91, 1.19) | 1.06 (95% CI: 0.93, 1.21) |
|  | Baseline to CVD | 1.03 (95% CI: 0.96, 1.12) | 1.04 (95% CI: 0.96, 1.12) | 1.03 (95% CI: 0.94, 1.12) | 1.03 (95% CI: 0.94, 1.13) | 1.03 (95% CI: 0.93, 1.14) |
|  | Cancer to CVD | 0.92 (95% CI: 0.75, 1.15) | 0.92 (95% CI: 0.74, 1.14) | 0.93 (95% CI: 0.75, 1.17) | 0.95 (95% CI: 0.77, 1.16) | 0.98 (95% CI: 0.82, 1.18) |
|  | T2D to CVD | 1.20 (95% CI: 1.09, 1.33) | 1.20 (95% CI: 1.09, 1.32) | 1.19 (95% CI: 1.08, 1.31) | 1.22 (95% CI: 1.10, 1.36) | 1.22 (95% CI: 1.09, 1.37) |
|  | Baseline to T2D | 1.13 (95% CI: 1.05, 1.21) | 1.13 (95% CI: 1.06, 1.21) | 1.14 (95% CI: 1.07, 1.23) | 1.16 (95% CI: 1.07, 1.24) | 1.17 (95% CI: 1.08, 1.26) |
|  | Cancer to T2D | 1.02 (95% CI: 0.88, 1.18) | 1.02 (95% CI: 0.88, 1.19) | 1.04 (95% CI: 0.90, 1.19) | 1.06 (95% CI: 0.93, 1.21) | 1.07 (95% CI: 0.93, 1.23) |
|  | CVD to T2D | 0.99 (95% CI: 0.75, 1.30) | 0.99 (95% CI: 0.74, 1.31) | 1.00 (95% CI: 0.76, 1.30) | 1.01 (95% CI: 0.78, 1.32) | 1.02 (95% CI: 0.81, 1.30) |
| SCLY | Baseline to Cancer | 1.15 (95% CI: 1.08, 1.23) | 1.17 (95% CI: 1.09, 1.25) | 1.14 (95% CI: 1.07, 1.22) | 1.14 (95% CI: 1.07, 1.22) | 1.14 (95% CI: 1.07, 1.22) |
|  | CVD to Cancer | 1.01 (95% CI: 0.87, 1.17) | 1.02 (95% CI: 0.88, 1.18) | 1.04 (95% CI: 0.89, 1.20) | 1.04 (95% CI: 0.90, 1.21) | 1.04 (95% CI: 0.89, 1.21) |
|  | T2D to Cancer | 1.28 (95% CI: 1.02, 1.60) | 1.27 (95% CI: 1.01, 1.59) | 1.28 (95% CI: 1.02, 1.60) | 1.28 (95% CI: 1.02, 1.60) | 1.30 (95% CI: 1.02, 1.66) |
|  | Baseline to CVD | 1.02 (95% CI: 0.93, 1.10) | 1.00 (95% CI: 0.92, 1.08) | 0.99 (95% CI: 0.92, 1.08) | 1.01 (95% CI: 0.93, 1.10) | 1.00 (95% CI: 0.92, 1.09) |
|  | Cancer to CVD | 0.93 (95% CI: 0.76, 1.14) | 0.93 (95% CI: 0.76, 1.14) | 0.93 (95% CI: 0.76, 1.14) | 0.94 (95% CI: 0.76, 1.15) | 0.91 (95% CI: 0.74, 1.13) |
|  | T2D to CVD | 1.15 (95% CI: 0.92, 1.44) | 1.14 (95% CI: 0.91, 1.43) | 1.13 (95% CI: 0.90, 1.42) | 1.12 (95% CI: 0.88, 1.42) | 1.08 (95% CI: 0.85, 1.38) |
|  | Baseline to T2D | 1.31 (95% CI: 1.22, 1.41) | 1.31 (95% CI: 1.22, 1.41) | 1.31 (95% CI: 1.21, 1.41) | 1.28 (95% CI: 1.19, 1.39) | 1.28 (95% CI: 1.19, 1.39) |
|  | Cancer to T2D | 1.56 (95% CI: 1.36, 1.80) | 1.56 (95% CI: 1.35, 1.79) | 1.57 (95% CI: 1.36, 1.81) | 1.56 (95% CI: 1.35, 1.80) | 1.59 (95% CI: 1.37, 1.84) |
|  | CVD to T2D | 1.35 (95% CI: 1.15, 1.59) | 1.35 (95% CI: 1.15, 1.59) | 1.35 (95% CI: 1.15, 1.59) | 1.32 (95% CI: 1.12, 1.56) | 1.32 (95% CI: 1.12, 1.56) |
| GALNT14 | Baseline to Cancer | 1.10 (95% CI: 1.03, 1.18) | 1.09 (95% CI: 1.02, 1.17) | 1.10 (95% CI: 1.02, 1.18) | 1.11 (95% CI: 1.03, 1.19) | 1.12 (95% CI: 1.04, 1.20) |
|  | CVD to Cancer | 0.94 (95% CI: 0.80, 1.11) | 0.93 (95% CI: 0.79, 1.10) | 0.95 (95% CI: 0.81, 1.12) | 0.95 (95% CI: 0.81, 1.12) | 0.94 (95% CI: 0.80, 1.11) |
|  | T2D to Cancer | 1.08 (95% CI: 0.83, 1.41) | 1.08 (95% CI: 0.83, 1.41) | 1.08 (95% CI: 0.82, 1.43) | 1.09 (95% CI: 0.83, 1.43) | 1.11 (95% CI: 0.85, 1.46) |
|  | Baseline to CVD | 1.03 (95% CI: 0.94, 1.12) | 1.04 (95% CI: 0.95, 1.13) | 1.03 (95% CI: 0.94, 1.13) | 1.05 (95% CI: 0.95, 1.15) | 1.04 (95% CI: 0.94, 1.15) |
|  | Cancer to CVD | 0.99 (95% CI: 0.78, 1.26) | 1.00 (95% CI: 0.79, 1.26) | 1.01 (95% CI: 0.80, 1.27) | 1.02 (95% CI: 0.81, 1.27) | 0.99 (95% CI: 0.80, 1.22) |
|  | T2D to CVD | 0.86 (95% CI: 0.68, 1.09) | 0.86 (95% CI: 0.68, 1.09) | 0.87 (95% CI: 0.68, 1.10) | 0.82 (95% CI: 0.64, 1.04) | 0.80 (95% CI: 0.62, 1.02) |
|  | Baseline to T2D | 1.09 (95% CI: 1.00, 1.20) | 1.10 (95% CI: 1.00, 1.21) | 1.10 (95% CI: 1.00, 1.21) | 1.11 (95% CI: 1.00, 1.23) | 1.10 (95% CI: 0.98, 1.23) |
|  | Cancer to T2D | 1.30 (95% CI: 1.18, 1.43) | 1.29 (95% CI: 1.17, 1.42) | 1.27 (95% CI: 1.15, 1.41) | 1.27 (95% CI: 1.15, 1.41) | 1.28 (95% CI: 1.15, 1.43) |
|  | CVD to T2D | 1.43 (95% CI: 0.96, 2.11) | 1.44 (95% CI: 0.97, 2.13) | 1.43 (95% CI: 0.96, 2.13) | 1.29 (95% CI: 0.88, 1.90) | 1.24 (95% CI: 0.84, 1.82) |
| ACY3 | Baseline to Cancer | 1.07 (95% CI: 1.01, 1.14) | 1.06 (95% CI: 1.00, 1.13) | 1.08 (95% CI: 1.02, 1.15) | 1.08 (95% CI: 1.02, 1.15) | 1.09 (95% CI: 1.03, 1.16) |
|  | CVD to Cancer | 0.91 (95% CI: 0.77, 1.07) | 0.92 (95% CI: 0.77, 1.08) | 0.91 (95% CI: 0.76, 1.08) | 0.90 (95% CI: 0.76, 1.08) | 0.89 (95% CI: 0.74, 1.07) |
|  | T2D to Cancer | 1.15 (95% CI: 0.98, 1.33) | 1.14 (95% CI: 0.97, 1.34) | 1.21 (95% CI: 1.08, 1.35) | 1.21 (95% CI: 1.08, 1.36) | 1.21 (95% CI: 1.07, 1.36) |
|  | Baseline to CVD | 1.10 (95% CI: 1.04, 1.17) | 1.09 (95% CI: 1.03, 1.17) | 1.10 (95% CI: 1.03, 1.17) | 1.10 (95% CI: 1.03, 1.18) | 1.11 (95% CI: 1.03, 1.19) |
|  | Cancer to CVD | 1.06 (95% CI: 0.91, 1.25) | 1.06 (95% CI: 0.91, 1.24) | 1.05 (95% CI: 0.90, 1.24) | 1.05 (95% CI: 0.91, 1.23) | 1.06 (95% CI: 0.91, 1.23) |
|  | T2D to CVD | 1.01 (95% CI: 0.75, 1.35) | 1.02 (95% CI: 0.76, 1.36) | 1.03 (95% CI: 0.78, 1.36) | 1.04 (95% CI: 0.79, 1.38) | 1.04 (95% CI: 0.78, 1.39) |
|  | Baseline to T2D | 1.20 (95% CI: 1.14, 1.27) | 1.20 (95% CI: 1.14, 1.27) | 1.21 (95% CI: 1.15, 1.28) | 1.20 (95% CI: 1.14, 1.28) | 1.20 (95% CI: 1.12, 1.27) |
|  | Cancer to T2D | 1.32 (95% CI: 1.16, 1.50) | 1.31 (95% CI: 1.15, 1.49) | 1.32 (95% CI: 1.16, 1.50) | 1.31 (95% CI: 1.15, 1.49) | 1.34 (95% CI: 1.14, 1.56) |
|  | CVD to T2D | 1.02 (95% CI: 0.76, 1.38) | 1.02 (95% CI: 0.76, 1.37) | 1.04 (95% CI: 0.78, 1.37) | 1.04 (95% CI: 0.77, 1.39) | 1.00 (95% CI: 0.72, 1.37) |
| RPN2 | Baseline to Cancer | 0.99 (95% CI: 0.92, 1.06) | 0.98 (95% CI: 0.91, 1.05) | 0.99 (95% CI: 0.92, 1.06) | 0.99 (95% CI: 0.92, 1.07) | 1.01 (95% CI: 0.93, 1.08) |
|  | CVD to Cancer | 1.02 (95% CI: 0.82, 1.26) | 1.04 (95% CI: 0.84, 1.29) | 1.07 (95% CI: 0.86, 1.32) | 1.07 (95% CI: 0.86, 1.32) | 1.06 (95% CI: 0.86, 1.32) |
|  | T2D to Cancer | 1.15 (95% CI: 0.93, 1.43) | 1.16 (95% CI: 0.93, 1.43) | 1.16 (95% CI: 0.94, 1.43) | 1.16 (95% CI: 0.93, 1.43) | 1.13 (95% CI: 0.92, 1.41) |
|  | Baseline to CVD | 1.12 (95% CI: 1.03, 1.21) | 1.11 (95% CI: 1.03, 1.20) | 1.10 (95% CI: 1.01, 1.19) | 1.10 (95% CI: 1.01, 1.20) | 1.09 (95% CI: 0.99, 1.20) |
|  | Cancer to CVD | 1.31 (95% CI: 1.00, 1.72) | 1.30 (95% CI: 1.00, 1.71) | 1.29 (95% CI: 0.98, 1.68) | 1.31 (95% CI: 1.00, 1.71) | 1.29 (95% CI: 0.98, 1.70) |
|  | T2D to CVD | 1.22 (95% CI: 0.91, 1.63) | 1.23 (95% CI: 0.90, 1.68) | 1.23 (95% CI: 0.91, 1.67) | 1.13 (95% CI: 0.89, 1.43) | 1.17 (95% CI: 0.92, 1.48) |
|  | Baseline to T2D | 1.14 (95% CI: 1.04, 1.25) | 1.15 (95% CI: 1.05, 1.26) | 1.15 (95% CI: 1.05, 1.27) | 1.16 (95% CI: 1.05, 1.27) | 1.16 (95% CI: 1.05, 1.28) |
|  | Cancer to T2D | 1.24 (95% CI: 0.91, 1.69) | 1.24 (95% CI: 0.90, 1.70) | 1.24 (95% CI: 0.89, 1.73) | 1.24 (95% CI: 0.89, 1.73) | 1.20 (95% CI: 0.84, 1.70) |
|  | CVD to T2D | 1.18 (95% CI: 0.78, 1.79) | 1.20 (95% CI: 0.78, 1.83) | 1.24 (95% CI: 0.81, 1.90) | 1.11 (95% CI: 0.71, 1.73) | 1.10 (95% CI: 0.70, 1.73) |
| CD209* | Baseline to Cancer | 1.00 (95% CI: 0.94, 1.07) | 1.01 (95% CI: 0.94, 1.08) | 1.01 (95% CI: 0.95, 1.09) | 1.02 (95% CI: 0.95, 1.10) | 1.03 (95% CI: 0.96, 1.12) |
|  | CVD to Cancer | 0.99 (95% CI: 0.82, 1.20) | 0.98 (95% CI: 0.81, 1.20) | 0.96 (95% CI: 0.79, 1.17) | 0.96 (95% CI: 0.78, 1.17) | 0.97 (95% CI: 0.79, 1.18) |
|  | T2D to Cancer | 0.94 (95% CI: 0.75, 1.18) | 0.95 (95% CI: 0.76, 1.18) | 0.93 (95% CI: 0.74, 1.16) | 0.93 (95% CI: 0.74, 1.16) | 0.96 (95% CI: 0.77, 1.19) |
|  | Baseline to CVD | 0.87 (95% CI: 0.80, 0.95) | 0.88 (95% CI: 0.81, 0.96) | 0.89 (95% CI: 0.81, 0.97) | 0.89 (95% CI: 0.81, 0.98) | 0.90 (95% CI: 0.82, 0.99) |
|  | Cancer to CVD | 1.04 (95% CI: 0.80, 1.35) | 1.02 (95% CI: 0.78, 1.34) | 1.03 (95% CI: 0.79, 1.34) | 1.03 (95% CI: 0.80, 1.35) | 1.06 (95% CI: 0.82, 1.37) |
|  | T2D to CVD | 1.08 (95% CI: 0.85, 1.36) | 1.07 (95% CI: 0.85, 1.36) | 1.07 (95% CI: 0.85, 1.35) | 1.05 (95% CI: 0.81, 1.35) | 1.06 (95% CI: 0.83, 1.37) |
|  | Baseline to T2D | 0.90 (95% CI: 0.82, 0.99) | 0.91 (95% CI: 0.82, 1.00) | 0.90 (95% CI: 0.81, 0.99) | 0.90 (95% CI: 0.81, 0.99) | 0.90 (95% CI: 0.81, 1.00) |
|  | Cancer to T2D | 1.03 (95% CI: 0.71, 1.48) | 1.03 (95% CI: 0.71, 1.50) | 1.03 (95% CI: 0.70, 1.50) | 1.03 (95% CI: 0.69, 1.53) | 1.00 (95% CI: 0.65, 1.54) |
|  | CVD to T2D | 1.41 (95% CI: 1.17, 1.71) | 1.40 (95% CI: 1.16, 1.68) | 1.37 (95% CI: 1.14, 1.65) | 1.32 (95% CI: 1.08, 1.60) | 1.30 (95% CI: 1.07, 1.59) |
| KCTD2 | Baseline to Cancer | 1.02 (95% CI: 0.93, 1.12) | 1.02 (95% CI: 0.93, 1.12) | 1.01 (95% CI: 0.92, 1.12) | 1.02 (95% CI: 0.92, 1.12) | 1.00 (95% CI: 0.90, 1.11) |
|  | CVD to Cancer | 0.90 (95% CI: 0.73, 1.10) | 0.90 (95% CI: 0.73, 1.11) | 0.91 (95% CI: 0.74, 1.13) | 0.91 (95% CI: 0.74, 1.13) | 0.91 (95% CI: 0.73, 1.13) |
|  | T2D to Cancer | 1.15 (95% CI: 0.92, 1.44) | 1.15 (95% CI: 0.91, 1.45) | 1.17 (95% CI: 0.93, 1.46) | 1.17 (95% CI: 0.93, 1.47) | 1.10 (95% CI: 0.89, 1.37) |
|  | Baseline to CVD | 1.09 (95% CI: 1.00, 1.18) | 1.08 (95% CI: 0.99, 1.17) | 1.07 (95% CI: 0.98, 1.16) | 1.04 (95% CI: 0.95, 1.15) | 1.04 (95% CI: 0.95, 1.15) |
|  | Cancer to CVD | 1.21 (95% CI: 1.04, 1.42) | 1.21 (95% CI: 1.03, 1.42) | 1.18 (95% CI: 0.99, 1.39) | 1.21 (95% CI: 1.03, 1.42) | 1.20 (95% CI: 1.01, 1.42) |
|  | T2D to CVD | 1.32 (95% CI: 1.15, 1.50) | 1.32 (95% CI: 1.15, 1.50) | 1.31 (95% CI: 1.15, 1.50) | 1.30 (95% CI: 1.13, 1.49) | 1.30 (95% CI: 1.13, 1.49) |
|  | Baseline to T2D | 1.13 (95% CI: 1.04, 1.23) | 1.14 (95% CI: 1.05, 1.23) | 1.14 (95% CI: 1.04, 1.24) | 1.11 (95% CI: 1.02, 1.21) | 1.12 (95% CI: 1.02, 1.23) |
|  | Cancer to T2D | 1.26 (95% CI: 1.07, 1.48) | 1.29 (95% CI: 1.09, 1.52) | 1.31 (95% CI: 1.11, 1.55) | 1.28 (95% CI: 1.08, 1.52) | 1.27 (95% CI: 1.05, 1.53) |
|  | CVD to T2D | 0.93 (95% CI: 0.67, 1.29) | 0.94 (95% CI: 0.68, 1.31) | 0.96 (95% CI: 0.69, 1.33) | 0.86 (95% CI: 0.61, 1.21) | 0.86 (95% CI: 0.60, 1.23) |
| PIPOX | Baseline to Cancer | 1.13 (95% CI: 1.06, 1.20) | 1.14 (95% CI: 1.06, 1.21) | 1.13 (95% CI: 1.05, 1.20) | 1.12 (95% CI: 1.05, 1.20) | 1.13 (95% CI: 1.05, 1.21) |
|  | CVD to Cancer | 0.87 (95% CI: 0.70, 1.09) | 0.87 (95% CI: 0.70, 1.09) | 0.88 (95% CI: 0.71, 1.10) | 0.90 (95% CI: 0.72, 1.11) | 0.90 (95% CI: 0.72, 1.13) |
|  | T2D to Cancer | 0.88 (95% CI: 0.69, 1.13) | 0.88 (95% CI: 0.69, 1.12) | 0.89 (95% CI: 0.70, 1.12) | 0.89 (95% CI: 0.71, 1.12) | 0.89 (95% CI: 0.70, 1.13) |
|  | Baseline to CVD | 1.07 (95% CI: 0.99, 1.15) | 1.07 (95% CI: 1.00, 1.15) | 1.07 (95% CI: 0.99, 1.15) | 1.07 (95% CI: 1.00, 1.16) | 1.06 (95% CI: 0.98, 1.15) |
|  | Cancer to CVD | 0.80 (95% CI: 0.57, 1.13) | 0.80 (95% CI: 0.56, 1.14) | 0.77 (95% CI: 0.53, 1.11) | 0.76 (95% CI: 0.52, 1.12) | 0.74 (95% CI: 0.49, 1.10) |
|  | T2D to CVD | 0.98 (95% CI: 0.80, 1.20) | 0.98 (95% CI: 0.80, 1.20) | 0.97 (95% CI: 0.80, 1.18) | 0.96 (95% CI: 0.77, 1.19) | 0.95 (95% CI: 0.76, 1.18) |
|  | Baseline to T2D | 1.15 (95% CI: 1.08, 1.24) | 1.15 (95% CI: 1.07, 1.24) | 1.15 (95% CI: 1.07, 1.24) | 1.14 (95% CI: 1.05, 1.23) | 1.12 (95% CI: 1.03, 1.21) |
|  | Cancer to T2D | 1.31 (95% CI: 1.14, 1.51) | 1.30 (95% CI: 1.13, 1.50) | 1.32 (95% CI: 1.14, 1.52) | 1.33 (95% CI: 1.15, 1.54) | 1.33 (95% CI: 1.15, 1.54) |
|  | CVD to T2D | 1.15 (95% CI: 0.89, 1.48) | 1.14 (95% CI: 0.89, 1.48) | 1.14 (95% CI: 0.87, 1.48) | 1.16 (95% CI: 0.89, 1.51) | 1.16 (95% CI: 0.89, 1.51) |
| TTC36 | Baseline to Cancer | 1.00 (95% CI: 0.94, 1.07) | 1.01 (95% CI: 0.94, 1.08) | 1.01 (95% CI: 0.94, 1.08) | 1.01 (95% CI: 0.94, 1.08) | 1.01 (95% CI: 0.94, 1.09) |
|  | CVD to Cancer | 1.10 (95% CI: 0.97, 1.25) | 1.11 (95% CI: 0.99, 1.26) | 1.12 (95% CI: 1.00, 1.26) | 1.12 (95% CI: 0.99, 1.26) | 1.10 (95% CI: 0.97, 1.26) |
|  | T2D to Cancer | 1.35 (95% CI: 1.16, 1.58) | 1.34 (95% CI: 1.15, 1.56) | 1.37 (95% CI: 1.20, 1.56) | 1.35 (95% CI: 1.18, 1.54) | 1.35 (95% CI: 1.19, 1.53) |
|  | Baseline to CVD | 1.00 (95% CI: 0.93, 1.08) | 1.02 (95% CI: 0.95, 1.10) | 1.02 (95% CI: 0.94, 1.10) | 1.01 (95% CI: 0.93, 1.09) | 0.99 (95% CI: 0.91, 1.08) |
|  | Cancer to CVD | 1.10 (95% CI: 0.91, 1.33) | 1.07 (95% CI: 0.88, 1.30) | 1.05 (95% CI: 0.86, 1.29) | 1.08 (95% CI: 0.88, 1.33) | 1.08 (95% CI: 0.88, 1.34) |
|  | T2D to CVD | 0.87 (95% CI: 0.62, 1.21) | 0.86 (95% CI: 0.61, 1.20) | 0.83 (95% CI: 0.60, 1.17) | 0.82 (95% CI: 0.58, 1.17) | 0.79 (95% CI: 0.55, 1.13) |
|  | Baseline to T2D | 1.19 (95% CI: 1.11, 1.28) | 1.21 (95% CI: 1.13, 1.29) | 1.21 (95% CI: 1.13, 1.29) | 1.21 (95% CI: 1.13, 1.29) | 1.20 (95% CI: 1.11, 1.28) |
|  | Cancer to T2D | 1.26 (95% CI: 1.04, 1.53) | 1.27 (95% CI: 1.05, 1.53) | 1.25 (95% CI: 1.03, 1.53) | 1.24 (95% CI: 1.02, 1.52) | 1.21 (95% CI: 0.97, 1.51) |
|  | CVD to T2D | 0.91 (95% CI: 0.77, 1.07) | 0.92 (95% CI: 0.77, 1.08) | 0.92 (95% CI: 0.78, 1.09) | 0.90 (95% CI: 0.75, 1.07) | 0.87 (95% CI: 0.71, 1.07) |
| EPS8L2 | Baseline to Cancer | 1.14 (95% CI: 1.06, 1.23) | 1.14 (95% CI: 1.05, 1.23) | 1.14 (95% CI: 1.05, 1.24) | 1.14 (95% CI: 1.05, 1.24) | 1.14 (95% CI: 1.04, 1.24) |
|  | CVD to Cancer | 1.10 (95% CI: 0.91, 1.33) | 1.08 (95% CI: 0.88, 1.31) | 1.08 (95% CI: 0.89, 1.32) | 1.07 (95% CI: 0.88, 1.30) | 1.05 (95% CI: 0.87, 1.28) |
|  | T2D to Cancer | 1.10 (95% CI: 0.92, 1.31) | 1.09 (95% CI: 0.91, 1.30) | 1.10 (95% CI: 0.92, 1.31) | 1.11 (95% CI: 0.93, 1.32) | 1.06 (95% CI: 0.90, 1.24) |
|  | Baseline to CVD | 1.01 (95% CI: 0.92, 1.11) | 1.02 (95% CI: 0.93, 1.12) | 1.02 (95% CI: 0.92, 1.13) | 1.05 (95% CI: 0.95, 1.16) | 1.05 (95% CI: 0.94, 1.17) |
|  | Cancer to CVD | 1.11 (95% CI: 0.87, 1.41) | 1.11 (95% CI: 0.87, 1.42) | 1.11 (95% CI: 0.87, 1.43) | 1.10 (95% CI: 0.84, 1.44) | 1.12 (95% CI: 0.85, 1.47) |
|  | T2D to CVD | 0.98 (95% CI: 0.79, 1.21) | 0.98 (95% CI: 0.79, 1.21) | 0.98 (95% CI: 0.80, 1.21) | 0.96 (95% CI: 0.76, 1.21) | 0.95 (95% CI: 0.75, 1.21) |
|  | Baseline to T2D | 1.11 (95% CI: 1.02, 1.21) | 1.11 (95% CI: 1.01, 1.21) | 1.09 (95% CI: 0.99, 1.19) | 1.07 (95% CI: 0.97, 1.18) | 1.06 (95% CI: 0.96, 1.17) |
|  | Cancer to T2D | 1.31 (95% CI: 1.12, 1.53) | 1.31 (95% CI: 1.11, 1.53) | 1.33 (95% CI: 1.14, 1.56) | 1.41 (95% CI: 1.17, 1.70) | 1.44 (95% CI: 1.20, 1.72) |
|  | CVD to T2D | 1.48 (95% CI: 1.16, 1.89) | 1.48 (95% CI: 1.17, 1.89) | 1.49 (95% CI: 1.17, 1.90) | 1.44 (95% CI: 1.11, 1.85) | 1.41 (95% CI: 1.10, 1.80) |
| LIMK1 | Baseline to Cancer | 0.89 (95% CI: 0.83, 0.96) | 0.90 (95% CI: 0.83, 0.97) | 0.92 (95% CI: 0.86, 0.99) | 0.92 (95% CI: 0.85, 0.99) | 0.91 (95% CI: 0.84, 0.98) |
|  | CVD to Cancer | 1.04 (95% CI: 0.84, 1.28) | 1.05 (95% CI: 0.86, 1.29) | 1.06 (95% CI: 0.86, 1.30) | 1.05 (95% CI: 0.85, 1.29) | 1.08 (95% CI: 0.88, 1.31) |
|  | T2D to Cancer | 1.23 (95% CI: 1.09, 1.39) | 1.23 (95% CI: 1.09, 1.39) | 1.23 (95% CI: 1.10, 1.39) | 1.23 (95% CI: 1.10, 1.38) | 1.23 (95% CI: 1.10, 1.38) |
|  | Baseline to CVD | 1.04 (95% CI: 0.96, 1.12) | 1.02 (95% CI: 0.94, 1.11) | 1.02 (95% CI: 0.94, 1.12) | 0.98 (95% CI: 0.90, 1.07) | 0.97 (95% CI: 0.88, 1.06) |
|  | Cancer to CVD | 1.51 (95% CI: 1.14, 2.00) | 1.55 (95% CI: 1.16, 2.07) | 1.53 (95% CI: 1.14, 2.05) | 1.60 (95% CI: 1.22, 2.10) | 1.62 (95% CI: 1.23, 2.13) |
|  | T2D to CVD | 1.13 (95% CI: 0.97, 1.32) | 1.13 (95% CI: 0.97, 1.32) | 1.12 (95% CI: 0.96, 1.32) | 1.13 (95% CI: 0.97, 1.33) | 1.13 (95% CI: 0.96, 1.33) |
|  | Baseline to T2D | 1.09 (95% CI: 1.00, 1.19) | 1.10 (95% CI: 1.01, 1.19) | 1.10 (95% CI: 1.01, 1.19) | 1.09 (95% CI: 1.00, 1.19) | 1.11 (95% CI: 1.02, 1.21) |
|  | Cancer to T2D | 1.05 (95% CI: 0.73, 1.50) | 1.05 (95% CI: 0.73, 1.50) | 1.03 (95% CI: 0.70, 1.54) | 1.01 (95% CI: 0.67, 1.53) | 0.96 (95% CI: 0.61, 1.52) |
|  | CVD to T2D | 1.18 (95% CI: 0.89, 1.57) | 1.19 (95% CI: 0.89, 1.58) | 1.21 (95% CI: 0.92, 1.60) | 1.15 (95% CI: 0.86, 1.53) | 1.11 (95% CI: 0.82, 1.51) |
| CYP3A4 | Baseline to Cancer | 0.98 (95% CI: 0.91, 1.06) | 1.00 (95% CI: 0.92, 1.08) | 1.00 (95% CI: 0.92, 1.08) | 0.99 (95% CI: 0.91, 1.08) | 0.98 (95% CI: 0.90, 1.07) |
|  | CVD to Cancer | 0.95 (95% CI: 0.77, 1.17) | 0.96 (95% CI: 0.78, 1.19) | 0.98 (95% CI: 0.79, 1.22) | 1.00 (95% CI: 0.81, 1.24) | 1.03 (95% CI: 0.82, 1.28) |
|  | T2D to Cancer | 1.07 (95% CI: 0.83, 1.37) | 1.05 (95% CI: 0.82, 1.34) | 1.06 (95% CI: 0.82, 1.35) | 1.06 (95% CI: 0.83, 1.35) | 1.05 (95% CI: 0.82, 1.34) |
|  | Baseline to CVD | 1.32 (95% CI: 1.22, 1.43) | 1.32 (95% CI: 1.21, 1.43) | 1.29 (95% CI: 1.19, 1.41) | 1.27 (95% CI: 1.16, 1.39) | 1.28 (95% CI: 1.16, 1.40) |
|  | Cancer to CVD | 1.53 (95% CI: 1.23, 1.92) | 1.55 (95% CI: 1.24, 1.94) | 1.52 (95% CI: 1.22, 1.90) | 1.56 (95% CI: 1.24, 1.96) | 1.53 (95% CI: 1.21, 1.93) |
|  | T2D to CVD | 1.94 (95% CI: 1.48, 2.56) | 1.93 (95% CI: 1.47, 2.53) | 1.89 (95% CI: 1.45, 2.47) | 1.90 (95% CI: 1.44, 2.49) | 1.81 (95% CI: 1.40, 2.34) |
|  | Baseline to T2D | 1.33 (95% CI: 1.21, 1.46) | 1.33 (95% CI: 1.21, 1.46) | 1.35 (95% CI: 1.22, 1.49) | 1.32 (95% CI: 1.19, 1.46) | 1.30 (95% CI: 1.18, 1.45) |
|  | Cancer to T2D | 1.19 (95% CI: 0.86, 1.65) | 1.21 (95% CI: 0.87, 1.67) | 1.21 (95% CI: 0.87, 1.68) | 1.20 (95% CI: 0.86, 1.67) | 1.08 (95% CI: 0.78, 1.49) |
|  | CVD to T2D | 1.05 (95% CI: 0.72, 1.52) | 1.04 (95% CI: 0.72, 1.50) | 1.04 (95% CI: 0.72, 1.52) | 0.98 (95% CI: 0.67, 1.42) | 0.95 (95% CI: 0.65, 1.38) |
| THBS2* | Baseline to Cancer | 1.15 (95% CI: 1.07, 1.23) | 1.14 (95% CI: 1.06, 1.23) | 1.14 (95% CI: 1.05, 1.22) | 1.12 (95% CI: 1.04, 1.22) | 1.11 (95% CI: 1.02, 1.20) |
|  | CVD to Cancer | 0.93 (95% CI: 0.78, 1.13) | 0.92 (95% CI: 0.76, 1.11) | 0.91 (95% CI: 0.75, 1.11) | 0.93 (95% CI: 0.77, 1.12) | 0.92 (95% CI: 0.75, 1.13) |
|  | T2D to Cancer | 1.08 (95% CI: 0.91, 1.28) | 1.07 (95% CI: 0.90, 1.27) | 1.08 (95% CI: 0.91, 1.28) | 1.08 (95% CI: 0.91, 1.29) | 1.09 (95% CI: 0.90, 1.31) |
|  | Baseline to CVD | 1.14 (95% CI: 1.05, 1.25) | 1.16 (95% CI: 1.06, 1.26) | 1.17 (95% CI: 1.07, 1.28) | 1.18 (95% CI: 1.08, 1.30) | 1.20 (95% CI: 1.09, 1.32) |
|  | Cancer to CVD | 1.14 (95% CI: 0.90, 1.45) | 1.13 (95% CI: 0.89, 1.45) | 1.16 (95% CI: 0.91, 1.48) | 1.16 (95% CI: 0.90, 1.49) | 1.13 (95% CI: 0.87, 1.48) |
|  | T2D to CVD | 0.95 (95% CI: 0.77, 1.16) | 0.95 (95% CI: 0.78, 1.16) | 0.95 (95% CI: 0.77, 1.16) | 0.96 (95% CI: 0.78, 1.17) | 0.95 (95% CI: 0.77, 1.17) |
|  | Baseline to T2D | 1.45 (95% CI: 1.34, 1.57) | 1.44 (95% CI: 1.33, 1.57) | 1.45 (95% CI: 1.33, 1.57) | 1.41 (95% CI: 1.29, 1.54) | 1.40 (95% CI: 1.28, 1.53) |
|  | Cancer to T2D | 1.97 (95% CI: 1.56, 2.48) | 1.96 (95% CI: 1.55, 2.47) | 2.03 (95% CI: 1.60, 2.57) | 2.00 (95% CI: 1.58, 2.53) | 1.99 (95% CI: 1.58, 2.51) |
|  | CVD to T2D | 1.09 (95% CI: 0.81, 1.48) | 1.08 (95% CI: 0.80, 1.46) | 1.06 (95% CI: 0.78, 1.44) | 1.01 (95% CI: 0.73, 1.39) | 1.03 (95% CI: 0.74, 1.43) |
| SIGLEC1 | Baseline to Cancer | 1.10 (95% CI: 1.04, 1.17) | 1.09 (95% CI: 1.02, 1.16) | 1.08 (95% CI: 1.02, 1.16) | 1.08 (95% CI: 1.00, 1.16) | 1.08 (95% CI: 0.99, 1.17) |
|  | CVD to Cancer | 1.13 (95% CI: 1.01, 1.27) | 1.13 (95% CI: 1.00, 1.26) | 1.13 (95% CI: 1.01, 1.26) | 1.13 (95% CI: 1.02, 1.26) | 1.12 (95% CI: 1.01, 1.25) |
|  | T2D to Cancer | 0.94 (95% CI: 0.73, 1.22) | 0.93 (95% CI: 0.72, 1.21) | 0.94 (95% CI: 0.72, 1.22) | 0.94 (95% CI: 0.73, 1.23) | 0.93 (95% CI: 0.70, 1.22) |
|  | Baseline to CVD | 1.07 (95% CI: 0.98, 1.16) | 1.07 (95% CI: 0.98, 1.17) | 1.06 (95% CI: 0.96, 1.17) | 1.10 (95% CI: 1.00, 1.20) | 1.10 (95% CI: 1.00, 1.21) |
|  | Cancer to CVD | 0.92 (95% CI: 0.63, 1.33) | 0.87 (95% CI: 0.58, 1.31) | 0.86 (95% CI: 0.56, 1.32) | 0.84 (95% CI: 0.54, 1.32) | 0.75 (95% CI: 0.46, 1.23) |
|  | T2D to CVD | 1.19 (95% CI: 0.89, 1.60) | 1.19 (95% CI: 0.88, 1.62) | 1.18 (95% CI: 0.86, 1.61) | 1.16 (95% CI: 0.84, 1.62) | 1.17 (95% CI: 0.84, 1.63) |
|  | Baseline to T2D | 1.08 (95% CI: 0.99, 1.18) | 1.08 (95% CI: 0.98, 1.18) | 1.08 (95% CI: 0.98, 1.19) | 1.08 (95% CI: 0.97, 1.19) | 1.04 (95% CI: 0.93, 1.17) |
|  | Cancer to T2D | 1.36 (95% CI: 1.22, 1.51) | 1.36 (95% CI: 1.22, 1.51) | 1.37 (95% CI: 1.23, 1.54) | 1.37 (95% CI: 1.22, 1.53) | 1.35 (95% CI: 1.19, 1.53) |
|  | CVD to T2D | 0.80 (95% CI: 0.56, 1.15) | 0.81 (95% CI: 0.56, 1.17) | 0.76 (95% CI: 0.51, 1.13) | 0.75 (95% CI: 0.49, 1.16) | 0.74 (95% CI: 0.47, 1.16) |
| GRN | Baseline to Cancer | 1.10 (95% CI: 1.02, 1.19) | 1.11 (95% CI: 1.03, 1.19) | 1.10 (95% CI: 1.02, 1.19) | 1.09 (95% CI: 1.01, 1.18) | 1.09 (95% CI: 1.01, 1.18) |
|  | CVD to Cancer | 1.21 (95% CI: 0.99, 1.48) | 1.21 (95% CI: 0.98, 1.48) | 1.21 (95% CI: 0.98, 1.49) | 1.20 (95% CI: 0.97, 1.48) | 1.23 (95% CI: 1.00, 1.53) |
|  | T2D to Cancer | 0.83 (95% CI: 0.66, 1.04) | 0.82 (95% CI: 0.66, 1.03) | 0.81 (95% CI: 0.65, 1.01) | 0.81 (95% CI: 0.65, 1.01) | 0.85 (95% CI: 0.68, 1.05) |
|  | Baseline to CVD | 1.04 (95% CI: 0.96, 1.13) | 1.07 (95% CI: 0.99, 1.16) | 1.07 (95% CI: 0.99, 1.16) | 1.09 (95% CI: 1.00, 1.19) | 1.09 (95% CI: 0.99, 1.19) |
|  | Cancer to CVD | 1.09 (95% CI: 0.91, 1.31) | 1.10 (95% CI: 0.93, 1.31) | 1.12 (95% CI: 0.94, 1.33) | 1.12 (95% CI: 0.94, 1.34) | 1.10 (95% CI: 0.91, 1.33) |
|  | T2D to CVD | 0.75 (95% CI: 0.60, 0.94) | 0.75 (95% CI: 0.60, 0.95) | 0.74 (95% CI: 0.60, 0.93) | 0.77 (95% CI: 0.62, 0.96) | 0.78 (95% CI: 0.63, 0.97) |
|  | Baseline to T2D | 1.02 (95% CI: 0.93, 1.12) | 1.02 (95% CI: 0.93, 1.12) | 1.02 (95% CI: 0.93, 1.12) | 1.01 (95% CI: 0.92, 1.12) | 1.01 (95% CI: 0.92, 1.12) |
|  | Cancer to T2D | 1.25 (95% CI: 1.04, 1.51) | 1.26 (95% CI: 1.05, 1.52) | 1.27 (95% CI: 1.05, 1.53) | 1.28 (95% CI: 1.07, 1.54) | 1.30 (95% CI: 1.09, 1.55) |
|  | CVD to T2D | 0.79 (95% CI: 0.56, 1.12) | 0.79 (95% CI: 0.56, 1.12) | 0.77 (95% CI: 0.54, 1.10) | 0.73 (95% CI: 0.50, 1.07) | 0.72 (95% CI: 0.49, 1.05) |
| FGFR1 | Baseline to Cancer | 0.98 (95% CI: 0.92, 1.06) | 0.98 (95% CI: 0.91, 1.05) | 0.99 (95% CI: 0.92, 1.06) | 0.98 (95% CI: 0.92, 1.06) | 1.00 (95% CI: 0.93, 1.08) |
|  | CVD to Cancer | 1.01 (95% CI: 0.83, 1.22) | 1.01 (95% CI: 0.83, 1.22) | 0.97 (95% CI: 0.81, 1.18) | 0.96 (95% CI: 0.80, 1.16) | 1.01 (95% CI: 0.84, 1.22) |
|  | T2D to Cancer | 0.85 (95% CI: 0.66, 1.09) | 0.83 (95% CI: 0.65, 1.07) | 0.84 (95% CI: 0.66, 1.08) | 0.84 (95% CI: 0.66, 1.08) | 0.89 (95% CI: 0.69, 1.14) |
|  | Baseline to CVD | 0.88 (95% CI: 0.80, 0.96) | 0.88 (95% CI: 0.80, 0.97) | 0.88 (95% CI: 0.80, 0.97) | 0.89 (95% CI: 0.81, 0.98) | 0.87 (95% CI: 0.79, 0.97) |
|  | Cancer to CVD | 1.00 (95% CI: 0.78, 1.29) | 1.01 (95% CI: 0.79, 1.29) | 0.99 (95% CI: 0.77, 1.27) | 0.99 (95% CI: 0.76, 1.28) | 1.01 (95% CI: 0.78, 1.31) |
|  | T2D to CVD | 0.63 (95% CI: 0.49, 0.82) | 0.64 (95% CI: 0.49, 0.82) | 0.63 (95% CI: 0.49, 0.81) | 0.64 (95% CI: 0.50, 0.81) | 0.63 (95% CI: 0.50, 0.80) |
|  | Baseline to T2D | 0.71 (95% CI: 0.64, 0.79) | 0.71 (95% CI: 0.64, 0.79) | 0.70 (95% CI: 0.63, 0.79) | 0.70 (95% CI: 0.63, 0.79) | 0.70 (95% CI: 0.62, 0.78) |
|  | Cancer to T2D | 0.82 (95% CI: 0.55, 1.22) | 0.83 (95% CI: 0.55, 1.23) | 0.85 (95% CI: 0.57, 1.29) | 0.86 (95% CI: 0.57, 1.31) | 0.91 (95% CI: 0.60, 1.38) |
|  | CVD to T2D | 0.63 (95% CI: 0.46, 0.88) | 0.64 (95% CI: 0.46, 0.89) | 0.65 (95% CI: 0.47, 0.91) | 0.72 (95% CI: 0.54, 0.97) | 0.73 (95% CI: 0.54, 0.97) |
| NXPH3 | Baseline to Cancer | 0.95 (95% CI: 0.88, 1.02) | 0.94 (95% CI: 0.88, 1.01) | 0.94 (95% CI: 0.87, 1.01) | 0.93 (95% CI: 0.86, 1.00) | 0.92 (95% CI: 0.86, 1.00) |
|  | CVD to Cancer | 0.86 (95% CI: 0.69, 1.06) | 0.85 (95% CI: 0.69, 1.06) | 0.86 (95% CI: 0.69, 1.07) | 0.89 (95% CI: 0.72, 1.10) | 0.88 (95% CI: 0.69, 1.11) |
|  | T2D to Cancer | 1.05 (95% CI: 0.93, 1.19) | 1.04 (95% CI: 0.92, 1.19) | 1.05 (95% CI: 0.93, 1.19) | 1.05 (95% CI: 0.93, 1.19) | 1.06 (95% CI: 0.93, 1.20) |
|  | Baseline to CVD | 1.03 (95% CI: 0.94, 1.11) | 1.03 (95% CI: 0.94, 1.12) | 1.02 (95% CI: 0.94, 1.11) | 1.01 (95% CI: 0.92, 1.10) | 1.01 (95% CI: 0.92, 1.11) |
|  | Cancer to CVD | 1.23 (95% CI: 0.99, 1.53) | 1.23 (95% CI: 0.99, 1.53) | 1.20 (95% CI: 0.97, 1.49) | 1.21 (95% CI: 0.96, 1.53) | 1.19 (95% CI: 0.93, 1.53) |
|  | T2D to CVD | 1.20 (95% CI: 1.09, 1.32) | 1.20 (95% CI: 1.09, 1.31) | 1.20 (95% CI: 1.09, 1.31) | 1.20 (95% CI: 1.10, 1.32) | 1.20 (95% CI: 1.09, 1.31) |
|  | Baseline to T2D | 1.28 (95% CI: 1.19, 1.38) | 1.30 (95% CI: 1.20, 1.40) | 1.30 (95% CI: 1.20, 1.41) | 1.29 (95% CI: 1.19, 1.40) | 1.32 (95% CI: 1.21, 1.44) |
|  | Cancer to T2D | 1.14 (95% CI: 0.90, 1.45) | 1.17 (95% CI: 0.92, 1.47) | 1.18 (95% CI: 0.92, 1.50) | 1.16 (95% CI: 0.91, 1.47) | 1.08 (95% CI: 0.83, 1.40) |
|  | CVD to T2D | 1.09 (95% CI: 0.82, 1.46) | 1.09 (95% CI: 0.83, 1.44) | 1.08 (95% CI: 0.82, 1.43) | 1.03 (95% CI: 0.74, 1.41) | 1.01 (95% CI: 0.73, 1.39) |
| SEMA7A | Baseline to Cancer | 1.13 (95% CI: 1.05, 1.21) | 1.14 (95% CI: 1.06, 1.22) | 1.13 (95% CI: 1.05, 1.21) | 1.11 (95% CI: 1.03, 1.20) | 1.12 (95% CI: 1.04, 1.21) |
|  | CVD to Cancer | 1.01 (95% CI: 0.85, 1.21) | 0.99 (95% CI: 0.83, 1.18) | 1.00 (95% CI: 0.83, 1.19) | 0.99 (95% CI: 0.83, 1.18) | 0.99 (95% CI: 0.83, 1.19) |
|  | T2D to Cancer | 0.99 (95% CI: 0.83, 1.19) | 0.99 (95% CI: 0.83, 1.19) | 0.98 (95% CI: 0.82, 1.17) | 0.99 (95% CI: 0.83, 1.18) | 1.03 (95% CI: 0.87, 1.23) |
|  | Baseline to CVD | 0.93 (95% CI: 0.85, 1.02) | 0.95 (95% CI: 0.87, 1.03) | 0.95 (95% CI: 0.86, 1.03) | 0.96 (95% CI: 0.87, 1.05) | 0.97 (95% CI: 0.88, 1.07) |
|  | Cancer to CVD | 1.07 (95% CI: 0.85, 1.34) | 1.04 (95% CI: 0.82, 1.32) | 1.02 (95% CI: 0.80, 1.30) | 1.00 (95% CI: 0.77, 1.29) | 0.95 (95% CI: 0.74, 1.23) |
|  | T2D to CVD | 0.85 (95% CI: 0.70, 1.04) | 0.85 (95% CI: 0.69, 1.04) | 0.84 (95% CI: 0.69, 1.03) | 0.80 (95% CI: 0.66, 0.98) | 0.79 (95% CI: 0.64, 0.96) |
|  | Baseline to T2D | 1.15 (95% CI: 1.05, 1.26) | 1.13 (95% CI: 1.04, 1.24) | 1.13 (95% CI: 1.03, 1.24) | 1.10 (95% CI: 1.00, 1.21) | 1.07 (95% CI: 0.97, 1.18) |
|  | Cancer to T2D | 1.54 (95% CI: 1.22, 1.94) | 1.56 (95% CI: 1.23, 1.97) | 1.58 (95% CI: 1.24, 2.00) | 1.60 (95% CI: 1.24, 2.06) | 1.62 (95% CI: 1.27, 2.07) |
|  | CVD to T2D | 1.30 (95% CI: 0.89, 1.88) | 1.30 (95% CI: 0.90, 1.89) | 1.26 (95% CI: 0.87, 1.83) | 1.29 (95% CI: 0.88, 1.90) | 1.23 (95% CI: 0.84, 1.81) |
| SEMA6A* | Baseline to Cancer | 1.15 (95% CI: 1.06, 1.25) | 1.16 (95% CI: 1.07, 1.26) | 1.17 (95% CI: 1.07, 1.28) | 1.17 (95% CI: 1.07, 1.28) | 1.16 (95% CI: 1.06, 1.27) |
|  | CVD to Cancer | 0.98 (95% CI: 0.84, 1.16) | 0.98 (95% CI: 0.83, 1.15) | 0.99 (95% CI: 0.84, 1.17) | 0.98 (95% CI: 0.83, 1.16) | 0.98 (95% CI: 0.83, 1.16) |
|  | T2D to Cancer | 1.09 (95% CI: 0.91, 1.30) | 1.09 (95% CI: 0.91, 1.30) | 1.08 (95% CI: 0.91, 1.28) | 1.09 (95% CI: 0.92, 1.30) | 1.12 (95% CI: 0.95, 1.33) |
|  | Baseline to CVD | 0.91 (95% CI: 0.82, 1.00) | 0.91 (95% CI: 0.82, 1.00) | 0.90 (95% CI: 0.81, 1.00) | 0.93 (95% CI: 0.84, 1.04) | 0.94 (95% CI: 0.84, 1.06) |
|  | Cancer to CVD | 1.04 (95% CI: 0.78, 1.40) | 1.01 (95% CI: 0.75, 1.36) | 1.00 (95% CI: 0.74, 1.34) | 0.98 (95% CI: 0.73, 1.32) | 0.99 (95% CI: 0.73, 1.34) |
|  | T2D to CVD | 0.94 (95% CI: 0.75, 1.18) | 0.94 (95% CI: 0.74, 1.18) | 0.93 (95% CI: 0.74, 1.18) | 0.90 (95% CI: 0.71, 1.13) | 0.88 (95% CI: 0.70, 1.10) |
|  | Baseline to T2D | 1.45 (95% CI: 1.29, 1.63) | 1.40 (95% CI: 1.24, 1.57) | 1.37 (95% CI: 1.22, 1.55) | 1.31 (95% CI: 1.16, 1.49) | 1.26 (95% CI: 1.11, 1.43) |
|  | Cancer to T2D | 2.54 (95% CI: 1.70, 3.78) | 2.59 (95% CI: 1.73, 3.89) | 2.45 (95% CI: 1.64, 3.66) | 2.47 (95% CI: 1.63, 3.76) | 2.42 (95% CI: 1.59, 3.68) |
|  | CVD to T2D | 1.30 (95% CI: 0.99, 1.70) | 1.30 (95% CI: 0.99, 1.69) | 1.28 (95% CI: 0.98, 1.67) | 1.26 (95% CI: 0.95, 1.67) | 1.25 (95% CI: 0.87, 1.80) |
| HNRNPAB* | Baseline to Cancer | 0.98 (95% CI: 0.92, 1.06) | 0.99 (95% CI: 0.92, 1.07) | 0.99 (95% CI: 0.92, 1.07) | 1.00 (95% CI: 0.93, 1.08) | 1.00 (95% CI: 0.92, 1.08) |
|  | CVD to Cancer | 1.09 (95% CI: 0.96, 1.24) | 1.09 (95% CI: 0.96, 1.24) | 1.09 (95% CI: 0.95, 1.24) | 1.09 (95% CI: 0.94, 1.26) | 1.08 (95% CI: 0.94, 1.24) |
|  | T2D to Cancer | 1.03 (95% CI: 0.84, 1.26) | 1.03 (95% CI: 0.84, 1.27) | 1.05 (95% CI: 0.85, 1.28) | 1.05 (95% CI: 0.86, 1.28) | 1.02 (95% CI: 0.82, 1.26) |
|  | Baseline to CVD | 1.14 (95% CI: 1.06, 1.24) | 1.12 (95% CI: 1.03, 1.22) | 1.13 (95% CI: 1.04, 1.23) | 1.10 (95% CI: 1.01, 1.21) | 1.09 (95% CI: 0.99, 1.21) |
|  | Cancer to CVD | 1.02 (95% CI: 0.71, 1.47) | 1.03 (95% CI: 0.72, 1.47) | 1.06 (95% CI: 0.76, 1.48) | 1.08 (95% CI: 0.77, 1.50) | 1.07 (95% CI: 0.76, 1.50) |
|  | T2D to CVD | 0.87 (95% CI: 0.69, 1.11) | 0.86 (95% CI: 0.68, 1.10) | 0.86 (95% CI: 0.67, 1.10) | 0.87 (95% CI: 0.68, 1.12) | 0.87 (95% CI: 0.67, 1.12) |
|  | Baseline to T2D | 1.03 (95% CI: 0.94, 1.12) | 1.04 (95% CI: 0.95, 1.13) | 1.03 (95% CI: 0.94, 1.13) | 1.04 (95% CI: 0.95, 1.15) | 1.05 (95% CI: 0.94, 1.16) |
|  | Cancer to T2D | 0.74 (95% CI: 0.49, 1.12) | 0.73 (95% CI: 0.48, 1.11) | 0.77 (95% CI: 0.51, 1.15) | 0.79 (95% CI: 0.53, 1.17) | 0.74 (95% CI: 0.48, 1.13) |
|  | CVD to T2D | 1.20 (95% CI: 1.04, 1.37) | 1.20 (95% CI: 1.02, 1.41) | 1.21 (95% CI: 1.04, 1.39) | 1.22 (95% CI: 1.07, 1.40) | 1.23 (95% CI: 1.10, 1.39) |
| KCNE5* | Baseline to Cancer | 0.91 (95% CI: 0.83, 0.98) | 0.90 (95% CI: 0.83, 0.98) | 0.90 (95% CI: 0.83, 0.98) | 0.90 (95% CI: 0.83, 0.99) | 0.90 (95% CI: 0.82, 0.99) |
|  | CVD to Cancer | 0.64 (95% CI: 0.50, 0.81) | 0.63 (95% CI: 0.49, 0.80) | 0.62 (95% CI: 0.49, 0.80) | 0.63 (95% CI: 0.49, 0.81) | 0.63 (95% CI: 0.49, 0.81) |
|  | T2D to Cancer | 0.80 (95% CI: 0.60, 1.05) | 0.79 (95% CI: 0.60, 1.05) | 0.80 (95% CI: 0.61, 1.06) | 0.80 (95% CI: 0.61, 1.06) | 0.85 (95% CI: 0.65, 1.11) |
|  | Baseline to CVD | 0.88 (95% CI: 0.80, 0.97) | 0.89 (95% CI: 0.80, 0.98) | 0.90 (95% CI: 0.81, 0.99) | 0.90 (95% CI: 0.81, 0.99) | 0.91 (95% CI: 0.82, 1.01) |
|  | Cancer to CVD | 0.97 (95% CI: 0.72, 1.30) | 1.00 (95% CI: 0.76, 1.33) | 0.96 (95% CI: 0.72, 1.26) | 0.97 (95% CI: 0.74, 1.28) | 0.97 (95% CI: 0.74, 1.27) |
|  | T2D to CVD | 0.83 (95% CI: 0.62, 1.13) | 0.85 (95% CI: 0.63, 1.14) | 0.86 (95% CI: 0.65, 1.15) | 0.88 (95% CI: 0.66, 1.17) | 0.93 (95% CI: 0.74, 1.15) |
|  | Baseline to T2D | 0.61 (95% CI: 0.51, 0.74) | 0.62 (95% CI: 0.51, 0.75) | 0.63 (95% CI: 0.52, 0.77) | 0.64 (95% CI: 0.52, 0.78) | 0.63 (95% CI: 0.53, 0.75) |
|  | Cancer to T2D | 0.53 (95% CI: 0.35, 0.78) | 0.53 (95% CI: 0.36, 0.78) | 0.54 (95% CI: 0.36, 0.80) | 0.51 (95% CI: 0.34, 0.77) | 0.54 (95% CI: 0.36, 0.82) |
|  | CVD to T2D | 0.86 (95% CI: 0.50, 1.47) | 0.86 (95% CI: 0.50, 1.49) | 0.86 (95% CI: 0.49, 1.52) | 0.92 (95% CI: 0.53, 1.62) | 0.92 (95% CI: 0.52, 1.63) |

## Partial R^2^ analyses for healthy lifestyle factors

| **Protein** | **Body mass index** | **Smoking** | **Alcohol** | **Physical activity** | **Diet** | **R^2^** |
| --- | --- | --- | --- | --- | --- | --- |
| ACAA1 | 0.45% | 0.04% | 1.80% | 0.01% | 0.02% | 7.61% |
| ACY3 | 0.02% | 0.08% | 0.12% | 0.00% | 0.00% | 2.12% |
| ATF6 | 1.96% | 0.73% | 0.53% | 0.26% | 0.20% | 20.83% |
| BMPER | 0.02% | 0.15% | 0.43% | 0.01% | 0.00% | 17.73% |
| CD209* | 0.38% | 0.14% | 0.01% | 0.09% | 0.01% | 5.40% |
| COA4 | 0.07% | 0.29% | 0.00% | 0.00% | 0.01% | 9.62% |
| CYP3A4 | 2.36% | 0.68% | 0.02% | 0.02% | 0.00% | 8.65% |
| DCPS | 0.30% | 0.15% | 0.17% | 0.03% | 0.10% | 13.65% |
| EPS8L2 | 0.70% | 0.09% | 0.26% | 0.01% | 0.02% | 19.97% |
| FABP1 | 0.07% | 0.08% | 0.10% | 0.00% | 0.02% | 12.73% |
| FGFR1 | 5.84% | 2.75% | 0.99% | 0.00% | 0.02% | 16.72% |
| GALNT14 | 0.08% | 0.07% | 0.70% | 0.01% | 0.09% | 13.71% |
| GC | 2.48% | 0.06% | 0.06% | 0.01% | 0.08% | 12.33% |
| GRN | 0.14% | 0.22% | 0.04% | 0.02% | 0.00% | 10.72% |
| HBG2 | 0.14% | 0.15% | 0.02% | 0.03% | 0.02% | 34.99% |
| HCAR2 | 1.27% | 0.05% | 0.01% | 0.00% | 0.00% | 9.35% |
| HIF1A | 0.20% | 0.12% | 0.02% | 0.00% | 0.00% | 3.48% |
| HMGCS1 | 1.91% | 0.79% | 0.49% | 0.04% | 0.08% | 21.57% |
| HNRNPAB* | 0.01% | 0.19% | 0.07% | 0.02% | 0.07% | 29.35% |
| HSPA6 | 1.34% | 0.16% | 0.00% | 0.00% | 0.01% | 4.49% |
| IL5RA | 0.02% | 0.15% | 0.00% | 0.00% | 0.01% | 8.73% |
| KCNE5* | 2.19% | 3.26% | 0.01% | 0.10% | 0.05% | 14.62% |
| KCTD2 | 0.05% | 0.12% | 0.01% | 0.01% | 0.01% | 6.77% |
| LIMK1 | 0.01% | 0.14% | 0.02% | 0.00% | 0.11% | 15.18% |
| NLGN1 | 1.81% | 0.39% | 0.35% | 0.02% | 0.03% | 22.93% |
| NXPH3 | 2.20% | 0.68% | 0.23% | 0.03% | 0.01% | 16.07% |
| PIPOX | 0.05% | 0.07% | 0.49% | 0.00% | 0.02% | 6.54% |
| RIDA | 6.40% | 0.24% | 0.73% | 0.05% | 0.11% | 15.31% |
| RNPEP | 0.04% | 0.29% | 0.11% | 0.06% | 0.05% | 19.36% |
| RPN2 | 1.23% | 0.18% | 0.01% | 0.00% | 0.01% | 3.76% |
| SCLY | 1.16% | 0.06% | 2.38% | 0.01% | 0.01% | 9.38% |
| SEMA6A* | 0.05% | 1.37% | 0.00% | 0.00% | 0.04% | 32.59% |
| SEMA7A | 0.02% | 1.01% | 0.01% | 0.00% | 0.01% | 20.52% |
| SIGLEC1 | 0.01% | 0.04% | 0.00% | 0.00% | 0.05% | 7.75% |
| SMOC1 | 0.77% | 2.22% | 0.54% | 0.01% | 0.01% | 22.33% |
| SNRPD2 | 0.34% | 0.12% | 0.00% | 0.00% | 0.04% | 28.37% |
| THBS2* | 1.54% | 0.10% | 0.01% | 0.01% | 0.14% | 17.72% |
| TTC36 | 1.61% | 0.11% | 0.15% | 0.01% | 0.00% | 4.92% |

## Pathways enrichment analysis

| **Category** | **Description** | **FDR value** |
| --- | --- | --- |
| GO Biological Process | Axon development | 7.80E-04 |
|  | Axon guidance | 0.0010 |
|  | Neuron projection development | 0.0010 |
|  | Cell adhesion | 0.0012 |
|  | Neurogenesis | 0.0012 |
|  | Generation of neurons | 0.0012 |
|  | Neuron differentiation | 0.0014 |
|  | Neuron development | 0.0020 |
|  | Cell morphogenesis | 0.0032 |
|  | Axonogenesis | 0.0032 |
|  | Neuron projection morphogenesis | 0.0032 |
|  | Locomotion | 0.0076 |
|  | Cell morphogenesis involved in differentiation | 0.0084 |
|  | Chemotaxis | 0.0089 |
|  | Plasma membrane bounded cell projection organization | 0.0089 |
|  | Cell development | 0.0166 |
|  | Nervous system development | 0.0317 |
|  | Enzyme-linked receptor protein signaling pathway | 0.0394 |
|  | Cellular component morphogenesis | 0.0394 |
|  | Positive regulation of cell migration | 0.0406 |
| GO Cellular Component | Extracellular region | 1.07E-06 |
|  | Cell surface | 9.58E-05 |
|  | Cell periphery | 9.58E-05 |
|  | Extracellular space | 1.00E-04 |
|  | Intrinsic component of membrane | 2.00E-04 |
|  | Anchored component of membrane | 0.0035 |
|  | Intrinsic component of plasma membrane | 0.0035 |
|  | Extracellular exosome | 0.0035 |
|  | Extracellular vesicle | 0.0035 |
|  | Plasma membrane | 0.0038 |
|  | Integral component of membrane | 0.0038 |
|  | Extracellular matrix | 0.0038 |
|  | Integral component of synaptic membrane | 0.0108 |
|  | Intrinsic component of synaptic membrane | 0.0110 |
|  | Glutamatergic synapse | 0.0276 |
|  | Collagen-containing extracellular matrix | 0.0307 |
|  | Membrane | 0.0319 |
|  | Receptor complex | 0.0319 |
|  | Intrinsic component of postsynaptic membrane | 0.0319 |
|  | Vesicle | 0.0386 |
| GO Molecular Function | Signaling receptor activity | 1.40E-04 |
|  | Transmembrane receptor protein tyrosine kinase activity | 0.0014 |
|  | Transmembrane receptor protein kinase activity | 0.0033 |
|  | Protein tyrosine kinase activity | 0.0197 |
|  | Transmembrane signaling receptor activity | 0.0473 |
| FDR: false discovery rate; GO: Gene Ontology | | |
